# Photoresponsive DNA steganography for secure information transmission by nanopore

**DOI:** 10.1093/nsr/nwag196

**Published:** 2026-04-01

**Authors:** Zheng-Li Hu, Jia Wang, Shao-Chuang Liu, Jia-Hong Wang, Hui Ma, Cheng Yang, Yi-Lun Ying, Feng Yan, Yi-Tao Long

**Affiliations:** Molecular Sensing and Imaging Center, School of Chemistry, Nanjing University, Nanjing 210023, China; Molecular Sensing and Imaging Center, School of Chemistry, Nanjing University, Nanjing 210023, China; Molecular Sensing and Imaging Center, School of Chemistry, Nanjing University, Nanjing 210023, China; Molecular Sensing and Imaging Center, School of Chemistry, Nanjing University, Nanjing 210023, China; Molecular Sensing and Imaging Center, School of Chemistry, Nanjing University, Nanjing 210023, China; School of Electronic Science and Engineering, Nanjing University, Nanjing 210023, China; Molecular Sensing and Imaging Center, School of Chemistry, Nanjing University, Nanjing 210023, China; Chemistry and Biomedicine Innovation Center, Nanjing University, Nanjing 210023, China; School of Electronic Science and Engineering, Nanjing University, Nanjing 210023, China; Molecular Sensing and Imaging Center, School of Chemistry, Nanjing University, Nanjing 210023, China

**Keywords:** photoresponsive DNA, aerolysin nanopore, single-molecule analysis, information encryption

## Abstract

Conventional DNA encryption methods often require additional noncoding strands as physical keys or covering media, leading to a density decrease from redundancy. Here, we present a nanopore-based photoresponsive DNA information steganography system (NAPDISS) that encodes 26 encrypted English letters by using only five isomerizable azobenzene-modified coding DNAs, thereby eliminating the need for extra synthesis. Encoding was implemented through two complementary schemes comprising a letter code with sequence-defined photoresponsive DNAs representing letters and an address code with poly(dA)_3_ (A3) concentrations defining the letters’ positions. Utilizing light as secret keys, NAPDISS conceals messages by obscuring the nanopore readouts, allowing recovery only through combined pre- and post-irradiation analyses. This approach achieves a logical storage density of 0.2–1.0 bits per nucleotide—about one order of magnitude higher than those of existing DNA-structure-based methods. Moreover, simplified sample processing reduces the readout time from hours to <10 minutes. Collectively, this work provides fresh insights into balancing density, security and efficiency, advancing DNA steganography toward secure and instantaneous messaging applications.

## Teaser

A nanopore-based photoresponsive DNA information-storage system (NAPDISS) that encodes and encrypts English text by using isomeric photoswitchable coding strands, thereby minimizing redundancy, enhancing storage density and improving readout efficiency.

## INTRODUCTION

Inspired by evolutionary natural selection, DNA has emerged as a promising medium for artificial data storage [1–4]. Information security remains a fundamental concern beyond the challenges of writing/reading costs, efficiency and accuracy [5–10]. Early studies introduced physical encryption strategies, such as concealing information-bearing DNAs within microdots [11] or everyday objects [12], to create complex steganographic environments for protecting secret messages. In these approaches, the intended message was extracted and decoded by using analysing polymerase chain reaction (PCR)-amplified DNA sequences. However, the information-leakage risk may increase with the advancement of sequencing technologies, particularly for coding strands that share common primer sequences. These concerns underscore the urgent need for more robust and reliable DNA encryption strategies to ensure information security.

Recent progress in DNA nanotechnology has facilitated information storage and secure transmission through the manipulation of programmable DNA structures [13–16]. In particular, the epigenetic information bits (epi-bit) storage framework integrates programmable DNA self-assembly with selective enzymatic methylation to write epigenetic modifications as information bits into DNA molecules [5]. By employing enzymatic epi-bit printing with prefabricated movable types and carriers, this approach enables the precise encoding of binary information onto universal DNA templates, providing a novel strategy for programmable and functional molecular data storage. DNA origami cryptography was proposed to encrypt information by using biotinylated message strands hybridized to the M13mp18 scaffold, forming origami structures recognizable by using an atomic force microscope after streptavidin binding [14]. More recently, DNA nanostructures of different shapes, numbers or sizes were employed to encode digital information, for instance by assembling dumbbell hairpins and multiway junctions on single-strand DNA (ssDNA) scaffolds [17,18]. In these works, information encryption and decryption are typically achieved via DNA strand displacement reactions, resulting in readout time extension (∼1 h), as accurate nanostructure folding requires labor-intensive sample preparation [17]. Notably, a minimum difference of 8 bp is necessary to distinguish the DNA assemblies used for binary encoding [19], which reduces the storage density by approximately two orders of magnitude. Although DNA-structure-based strategies demonstrate the feasibility of information encryption, low storage density remains a challenge for their use in secure information storage and communication.

Stimuli-responsive moieties can serve as programmable functional groups for information encryption via controlled structural changes, such as photoisomerizable azobenzene and light-cleavable *o*-nitroveratryl ether substituents [20–22]. Here, we introduce a nanopore-based photoresponsive DNA information steganography system (NAPDISS) that hides and obfuscates the target messages, relying on the controllable photoisomerization of five pre-synthesized azobenzene-modified coding DNAs (5 nt). This approach minimizes redundancy by eliminating the need for additional noncoding strands for information encryption, in turn, increasing the storage density. For rapid readout, subtle structural variations in photoisomerizable coding DNAs are directly identified by using aerolysin nanopores—one of the most sensitive biological nanopores for short ssDNA detection [23–25]. By employing different light-irradiation conditions as secret keys, NAPDISS protects the encrypted messages in a controllable and sustainable manner, allowing one cycle of information writing and reading to be completed within a few minutes. This strategy can be potentially adapted to other stimuli-responsive molecules for multidimensional encryption [16,26–30], opening up opportunities in biology, chemistry and material science to advance high-security and user-friendly molecular information technologies.

## RESULTS

**Figure 1.**
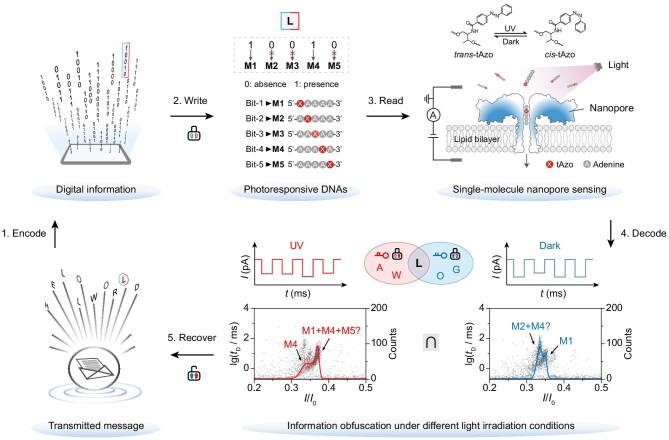
Workflow of the nanopore-based photoresponsive DNA information steganography system (NAPDISS). The read-write cycle comprises five main steps: (1) Encode: text characters are converted into binary codes according to the five-bit Baudot scheme (e.g. ‘L’ = 10010). (2) Write: binary information is stored in specific combinations of five azobenzene-modified DNA sequences (**M1**–**M5**), each corresponding to a defined bit position (Bit-1 to Bit-5). The presence of coding DNAs is denoted as ‘1’ and absence is denoted as ‘0’; thus ‘L’ is represented by a mixture of **M1** and **M4**. (3) Read: aerolysin nanopore sensing is used to analyze the photoresponsive DNA mixtures in individual samples under darkness either before (Dark) or after UV irradiation (UV), generating distinct single-molecule current signals due to photoinduced conformational changes. (4) Decode: reference nanopore current spectra of pure **M1**–**M5** components are employed to infer DNA identity, composition and the corresponding encoded bit values. Dark and UV analyses produce different obfuscated outputs. For example, dark readouts may yield ‘G (11010)’, ‘L (10010)’ and ‘O (11000)’, corresponding to combinations of **M1** and **M2**/**M4**, whereas UV readouts may produce ‘A (00011)’, ‘L (10010)’ and ‘W (10011)’, corresponding to combinations of **M4** and M**1**/**M5**. (5) Recover: the intended message is recovered by extracting the common output from the two candidate sets, with ‘L’ representing the correct character in the above example. No additional noncoding strands are needed, as information encryption is implemented by using different UV irradiation conditions (on/off) as secret keys: Dark for Key I and UV for Key II.

To build the reliable NAPDISS, five photoresponsive DNAs were fabricated to match with Bit-1 to Bit-5 of five-bit Baudot binary encoding, each of which incorporates an azobenzene as a pseudo nucleotide to polydeoxyadenine to form a coding strand (see Fig. 1 and Fig. S1). Azobenzene-modified DNA shows excellent photostability, long-term durability and sustained information retention, making it a promising molecular information-storage medium [31,32]. NAPDISS starts by encoding information into specific combinations of the five digital DNAs, in which the absence or presence of coding strands represent the corresponding bit as ‘0’ or ‘1’. In this way, the 26 letters of the English alphabet are written and encrypted by using five photoresponsive digital DNAs that can switch between two isomeric states regulated by light irradiation. Owing to the small pore diameter (∼1 nm) and strong spatial confinement of the aerolysin nanopore that enhance analyte interactions [23–25], these photoisomers will produce single-molecule signals with different current profiles when detected under darkness before (Dark) and after ultraviolet (UV, λ = 365 nm) irradiation. The typical signals contribute to different nanopore current spectra due to photo-modulated structural changes in the coding strands, which are analysed and then decoded into several possible obscuring results either with or without UV irradiation, suggesting the intrinsic capability of photoisomerizable DNAs for information encryption.

Intriguingly, the original information can be extracted and recovered only when combining and taking the intersection of the results under two different irradiation situations. Thus, the correct message is well protected by a pair of secret keys (Dark for Key I and UV for Key II). Using the DNA obfuscation and steganography (PDOS) strategy, secure information transmission can, in principle, be achieved for any text composed of English letters. The transmitted message cannot be deciphered and recovered by using conventional methods such as mass spectrometry and ultraviolet–visible absorption spectroscopy because all five photoresponsive coding DNAs have identical molecular weights and the same azobenzene chromophore (see Figs S2 and S3), highlighting the high-level information security of this well-designed system. This approach requires five pre-synthesized azobenzene-modified DNAs as movable blocks to encode all English letters (A–Z), unlike conventional methods that necessitate the continuous synthesis of encoding molecules for new messages and complementary strand displacement for information encryption. The reusable photoresponsive DNAs simplify the workflow by eliminating repetitive synthesis and enabling sample processing with a few minutes of light exposure. This strategy would advance molecular information encryption by balancing security, efficiency and sustainability.

### Design and detection of photoresponsive DNAs for information encryption

**Figure 2.**
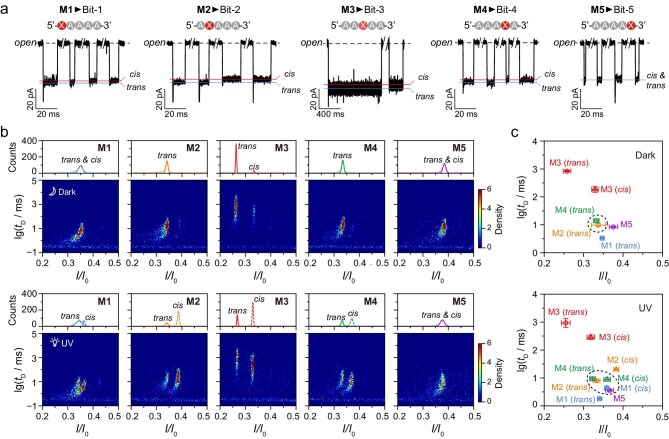
Design and detection of digital photoresponsive DNAs. (a) Sequences and typical signals of five digital photoresponsive DNAs for information encryption. The positionally isomeric **M1**–**M5** are designed to represent the corresponding Bit-1 to Bit-5 of the five-bit Baudot codes and detected by an aerolysin nanopore. Solid lines indicate the residual currents of the *trans*-tAzo (blue), the *cis*-tAzo (red) and the indiscernible (gray) photoisomers. (b) Current histograms and 2D density scatter plots of *I*/*I*_0_ vs. lg(*t*_D_) for the five photoresponsive digital DNAs before (upper) and after (lower) UV irradiation. (c) Comparison of *I*/*I*_0_ and lg(*t*_D_) for the detectable photoisomers before (upper) and after (lower) UV irradiation. The circles represent the *trans*-tAzo isomers and the triangles represent the *cis*-tAzo isomers. Dashed circles indicate the photoisomers exhibiting comparable current blockages and duration times. The final concentration of the five customized digital DNAs is fixed at 1.0 μM. Data were obtained at +120 mV in the buffer solution consisting of 1 M KCl, 10 mM Tris and 1 mM EDTA, pH 8.0.

Five digital DNAs were designed, namely **M1** (XA4), **M2** (AXA3), **M3** (A2XA2), **M4** (A3XA) and **M5** (A4X), each carrying an azobenzene moiety at a distinct position of poly(dA)_4_ via a threoninol linker (see Fig. 2a and Fig. S4). Aerolysin nanopore detection of **M1**–**M4** revealed deeper current blockages for the *trans*-tAzo isomers and shallower ones for the *cis*-tAzo isomers, with state assignments based on the well-established *trans*-to-*cis* photoisomerization of azobenzene under UV irradiation. **M5** produced signals with nearly identical current blockage due to unidentifiable isomeric conformations. The 5-nt ssDNA is fully accommodated within the sensing region of ∼24 Å (from R218 to N274) of the nonuniformly shaped aerolysin nanopore [33–35]. Variations in the position of the azobenzene moiety influence the characteristics of the ionic current blockages, most likely by altering the nanopore–analyte interactions within this confined sensing volume [36–38]. Typical signals of the five photoresponsive digital DNAs generated distinctly different distributions before and after UV irradiation, primarily due to modulations in the relative proportions of *trans*-tAzo and *cis*-tAzo regulated by the external irradiation (see Fig. 2b). As a control, the unmodified poly(dA)_5_ (A5) produced only one population, as its conformation remained fairly stable even under light illumination, indicating no light-induced change (see Fig. S5). In contrast, for **M3**, the *trans*-tAzo and the *cis*-tAzo populations are well defined either with or without irradiation, consistently with our previous work [25]. While no splitting populations were detected for **M5** regardless of the irradiation applied, for **M1, M2** and **M4**, the *trans*-tAzo populations were observed before irradiation, whereas the *cis*-tAzo populations appeared after UV irradiation when photoisomerization occurred.

The findings inspired us to use the possible obscuring nanopore readouts induced by multiple DNA photoisomers for information encryption. Herein, *I*_0_ represents the current for an open nanopore, *I* is the residual current of a nanopore blocked by single molecules, *I*/*I*_0_ is the residual current blockage and *t*_D_ is the duration time for a molecule staying inside a nanopore. Further analysis revealed that **M3** shows the longest *t*_D_ values and deepest current blockages either in the *trans*-tAzo or the *cis*-tAzo states, suggesting easy identification (see Fig. 2c and Fig. S6), whereas the other four digital DNAs have comparable duration times, making them difficult to distinguish. In the dark, before irradiation, larger *I*/*I*_0_ values of 0.35 ± 0.01 for **M1** and 0.38 ± 0.01 for **M5** are revealed, supporting direct identification of their *trans*-tAzo isomers (see Fig. 2c, upper). In contrast, **M2** and **M4** show close *I*/*I*_0_ values of ∼0.33 and similar *t*_D_ values of ∼10 ms for their *trans*-tAzo isomers, leading to obscuring of the results during discrimination. Interestingly, after UV irradiation, **M2** turns out to have the largest *I*/*I*_0_ value of 0.38 ± 0.01 for its *cis* isomers, thus accounting for the clear recognition (see Fig. 2c, lower). The overlapped current distributions result in uncertain determination of **M1, M4** and **M5** after UV irradiation (see Fig. S7). Consequently, the five photoresponsive digital DNAs could be readily identified by combining the nanopore results under both light-irradiation conditions and then the information could be decoded and fully recovered.

### Reading and decoding information in non-natural DNAs with biological nanopores

**Figure 3.**
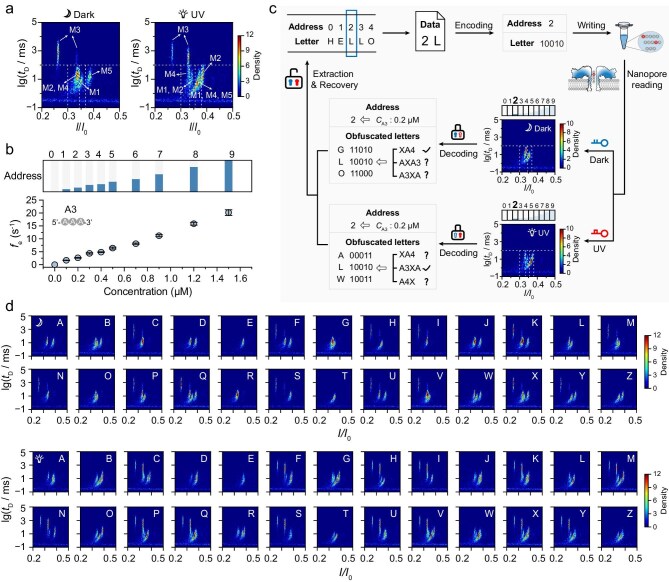
Nanopore-assisted information storage and encryption using photoresponsive DNAs. (a) 2D density scatter plots of premixed signals of five pure DNA samples before (left) and after (right) UV irradiation. Each plot was divided into several regions according to the duration and current distributions for the identification of **M1**–**M5**. Dashed lines indicate the thresholds of duration time and residual current blockages. (b) Address codes dependent on the effective frequencies (*f*_e_) of A3 at different concentrations. The *f*_e_ values increase linearly with A3 concentrations elevating from 0 to 1.5 μM and thus 10 concentration gradients were designed to represent the address codes ‘0’ to ‘9’. (c) Illustration of a general scheme for the storage and encryption of an addressable letter. As an example, the addressable ‘2L’ is written by using a mixture of **M1, M4** and A3, and then decoded with the assistance of a biological nanopore sensing approach. The correct message can only be unlocked by using double keys through a joint analysis to extract the intersection of the obscuring nanopore readouts before and after UV irradiation. Different light-irradiation conditions are adopted as the decryption keys, including dark before irradiation for Key I (blue) and after UV irradiation for Key II (red). (d) 2D density scatter plots for the 26 English letters before (upper) and after (lower) UV irradiation. Each plot was produced by using premixed signals of the corresponding digital DNAs. Typical signals (*N* > 1500) were collected for every pure sample at +120 mV.

To examine the feasibility of NAPDISS for information encryption, we firstly mixed the experimental data of independently tested samples to study the distinguishability of the customized digital DNAs (see Fig. 3a). The 2D scatter plots were divided into several regions, in which the threshold of *t*_D_ was defined as 100 ms and the thresholds of *I*/*I*_0_ were determined based on the current distributions (see Fig. S7). In the dark, without irradiation, the presence of **M3** could be easily found, as it is the only one that would produce signals with a duration time of >100 ms. Meanwhile, **M1** and **M5** could be identified via the gathered *trans*-tAzo populations based on different current distributions. However, **M2** and **M4** could not be readily recognized due to the indistinguishable current and duration distributions, providing a possibility for information obfuscation. After UV irradiation, the presence of **M2, M3** and **M4** could be clearly determined, while **M1** and **M5** would create obscuring results during information decoding. Recovery of an encrypted message cannot be achieved either before or after UV irradiation, as it is allowed only by combining the nanopore readouts under different light conditions. Therefore, confidential messages can be hidden in various obscuring pieces of information and decrypted only with double secret keys (Dark for Key I and UV for Key II).

To facilitate information recovery, we addressed every letter in a text with varying concentrations of poly(dA)_3_ (A3), the current distribution of which is completely separated from the coding DNAs (see Fig. 3b and Figs S8–S10). The effective frequencies (*f*_e_) of A3 were calculated to increase linearly to 20.16 ± 1.10 s^−1^ when the concentrations were increased to 1.5 μM when measured either individually or in a mixture (see Fig. S11). Therefore, the 10 increasing concentrations were used to indicate the address codes (letter numbers) ‘0’ to ‘9’. For example, the address code ‘0’ for the first letter is represented by A3 at a concentration of 0 μM and the following address code ‘1’ refers to A3 at a concentration of 0.1 μM. The concentration-dependent addressing system demonstrates scalable potential through the incorporation of additional molecular species and enhanced concentration resolution (see Figs S12 and S13). In this way, an addressable letter can be stored by using a mixture composed of A3 and the photoresponsive coding DNAs.

Taking the third letter ‘L’ in the word ‘HELLO’ as an example, its letter code and address code are ‘10010’ and ‘2’, thus written as a mixture containing **M1** and **M4** of equal concentration and A3 at a concentration of 0.2 μM (see Fig. 3c). After detection by the aerolysin nanopore, the concentration of A3 was estimated to be 0.2 μM compared with the well-established *f*_e_ standards (see Fig. S11). However, the statistical analysis revealed different results for the photoresponsive DNAs before and after irradiation. According to the identification scheme explained above, two populations were obtained with Key I (Dark), resulting in three possible outputs of ‘G’, ‘L’ and ‘O’ due to the positively identified **M1** and uncertain **M2** and **M4**. With Key II (UV), **M4** is clearly determined, while **M1** and **M5** remain unclear in the mixture, leading to the message being hidden in a set of obscuring codes ‘00011’ for ‘A’, ‘10010’ for ‘L’ and ‘10011’ for ‘W’. Finally, only with a combination of Key I and Key II can the original message ‘2L’ be deciphered and recovered by specifically taking the intersection of the two sets of obscuring letters. In this regard, NAPDISS operates as a double lock-and-key encryption system, which can be opened only with both keys and knowledge of the special method for data recovery, effectively protecting the secret message from potential interceptors. Theoretically, secure storage of the 26 English letters can be realized by using NAPDISS (see Fig. 3d and Figs S14 and S15).

### NAPDISS allows the steganography and transmission of encrypted messages

**Figure 4.**
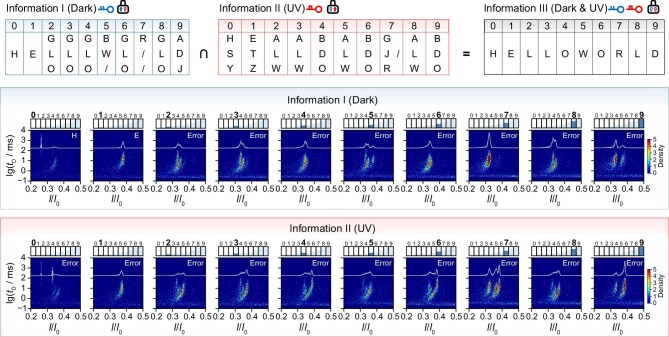
Implementation of secret-message transmission using NAPDISS. The decryption results of a 10-letter message and the related nanopore readouts. Information I and II are responsible for the message being decoded and recovered with Key I before irradiation and Key II after irradiation. The ciphertext stored in photoresponsive DNAs cannot be correctly decrypted by the interceptors with either Key I or Key II, and can only be fully recovered by the intended receiver with both keys. ‘Error’ indicates a confusing result that includes more than one possible obscuring output. Each letter is written by using a mixture composed of the photoresponsive DNAs of equal concentration and A3 at a specific concentration relevant to the address codes. For the current histogram and 2D density scatter plot of each DNA mixture, data were collected by using aerolysin nanopore during a single-channel recording of 5 minutes at +120 mV before or after UV irradiation.

To demonstrate the capability of NAPDISS for transmitting encrypted information, the text ‘HELLO’ ‘WORLD’ was used as the original information in a proof-of-concept experiment. In this scenario, the sender would transmit a secret message to the receiver, who shared the correct secret keys and data-recovery strategy (see Fig. 4). After receiving the DNA samples, the aerolysin nanopore detects them for information decoding and recovery by quantifying the concentration of A3 and recognizing the presence or absence of the five digital photoresponsive DNAs. Results that are not able to correctly decode into a unique code will output as ‘Error’. For Interceptor 1 with Key I (Dark), although the address code is well determined, the 10 letters cannot be completely decrypted due to the uncertain presence of **M2** and **M4** for defining the corresponding Bit-2 and Bit-4 (see Fig. S16). For example, the letter ‘W (10011)’ is likely misidentified as ‘B (11001)’ or ‘/(11011)’, where ‘/’ represents a five-bit code that does not correspond to any English letters. Likewise, for Interceptor 2 with Key II (UV), the ambiguity between **M1** and **M5** can lead to multiple molecular assignments, resulting in candidate output codes ‘W (10011)’, ‘L (10010)’ and ‘A (00011)’. Consequently, Interceptor 1 would acquire 6561 possible decryption results for the 10-letter message, whereas Interceptor 2 with Key II (UV) would obtain 78 732 confused letter combinations. Hence, the steganography strategy renders the encrypted message indistinguishable to interceptors, irrespective of the key applied. Eventually, the intended receiver, who possesses both decryption keys, would decipher and fully recover the desired message from the decoy messages only if performing a collaborative analysis to extract common outputs from Information I and Information II. As a result, the intended message ‘HELLO’ ‘WORLD’ was securely transferred from the sender to the receiver, proving the feasibility and reliability of NAPDISS for information transmission.

Unlike long-read sequencing that extracts native DNA sequence-based information [5,9,39], our approach encodes and decodes structure-based information in nonnaturally modified photoresponsive DNAs. By confining data recovery to precisely defined illumination parameters (e.g. specific wavelength and intensity), it provides robust security that is unattainable with native or naturally modified DNAs, including the methylated variants used in long-read sequencing scenarios. In contrast to DNA origami-based encryption strategies that require biotinylated complementary strands to assemble recognizable DNA structures [14,15,17], NAPDISS applies light-irradiation conditions as secret keys to conceal information within obscuring nanopore readouts of light-regulated isomeric DNAs (see Table S1). Our approach uses five modular 5-nt DNA sequences for programmable letter encoding using 5–25 nucleotides (e.g. ‘E’ = 00001, 5 nt; ‘L’ = 10010, 10 nt), thereby achieving an average logical storage density of 0.2–1.0 bits/nt, which is about one order of magnitude higher than those of conventional DNA-structure-based encryption methods [14,17,18,40,41]. Moreover, the sample processing is simplified, requiring only a few minutes of light irradiation for rapid information readout and decryption. In an optimized writing/reading cycle, the sample irradiation time can be reduced to 5 minutes (see Fig. S17) and a discernible 2D density scatter plot is obtainable from a 1-minute single-channel recording (see Figs S18–S21), thereby permitting information decoding and recovery within 10 minutes. As an alternative to conventional DNA encryption approaches [42], our strategy could improve information security by integrating more stringent parameters into the secret key. Using the letter ‘L’ as an example, three distinguishable populations can be found with UV (λ = 365 nm) irradiation of >5 minutes (see Fig. S17). However, the third population is not readily observed when either reducing the UV (λ = 365 nm) light intensity by half (see Fig. S22) or treating the samples with visible (λ = 533 nm) light (see Fig. S23), even after an extended irradiation of 15 minutes. These findings revealed that the information can only be decrypted by using UV (λ = 365 nm) light with sufficient intensity (e.g. ∼12 mW) and irradiation time (≥5 minutes). Beyond a simple UV light on/off stimulus, the secret key comprises multidimensional parameters that collectively define a moderately strict operational range that is narrow enough to prevent decoding under mismatched conditions yet reproducible under well-defined illumination, thereby ensuring information security and practical feasibility.

## DISCUSSION

This work demonstrated secure and efficient information transmission through DNA photoisomerization. We established an encryption system, NAPDISS, that uses five positionally isomeric photoresponsive DNAs for message encoding, while decryption is achieved via aerolysin nanopore analysis. Azobenzene-modified DNAs were carefully designed as the paradigm and they isomerize between the *trans*-tAzo and the *cis*-tAzo forms under controlled illumination, enabling simple one-step sample processing for rapid information recovery during the write–read cycle. NAPDISS employs different light-irradiation conditions as secret keys to hide messages in photoisomerizable coding strands, apparently reducing redundancy by eliminating the need for additional noncoding strands as covering media. The isomeric coding DNAs cannot be decrypted by using commonly used *α*-hemolysin (*α*-HL) and *Mycobacterium smegmatis* porin A (MspA) nanopores (see Figs S24–S26), aerolysin mutants (see Fig. S27) and other decoding techniques such as mass spectrometry and ultraviolet–visible absorption spectroscopy (see Figs S2 and S3). These results indicate that accurate decoding depends on both nanopore selection and precise light-irradiation parameters, underscoring the strong information security of this photoresponsive DNA storage system.

As information security, reading efficiency and storage density are effectively balanced, NAPDISS is promising for potential applications in secure and instant message communication. The nanopore detection of each sample for an encrypted character required ∼2 minutes in our proof-of-concept experiments. Future endeavors will further optimize the information-reading process and improve overall efficiency through the integration of a parallel detection strategy with a droplet nanopore array platform [43]. Scalability beyond the 26-letter alphabet can be achieved by increasing the encoding length to introduce additional bit positions and by diversifying molecular states through new chemical modifications. An automated end-to-end system is expected to advance DNA information encryption toward convenient applications, such as intelligent and portable devices integrating parallel nanopore sensing [43,44] and digital microfluidics [45,46], overcoming the limitations of single-pore detection and expanding the information capacity. A dedicated current-level-to-bit-value mapping database, supported by adaptive signal-analysis algorithms and models [47], is essential for automated signal classification and DNA component identification, thereby enabling accurate bit-value decoding and reliable information recovery. Besides DNA, other polymers composed of diverse man-made building blocks, such as abiotic peptides [48], poly(phosphodiester)s [49], oligo(amide-urethane)s [50] and oligourethanes, have been used as alternatives for digital information storage, while detection methods are limited to mass spectrometry. As a promising platform for detecting and decoding both biological and nonbiological informational polymers at the single-molecule level, NAPDISS shows great potential to pave the way for next-generation molecular information technology.

## Supplementary Material

nwag196_Supplemental_File
