# Supplementary material for: Photoresponsive DNA steganography for secure information transmission by nanopore
Source: Natl Sci Rev. 2026 Apr 1;13(9):nwag196. doi: 10.1093/nsr/nwag196 (PMC13185531; doi:10.1093/nsr/nwag196)
Supplement: nwag196_Supplemental_File [file nwag196_supplemental_file.pdf]

Supplementary Information for

Photoresponsive DNA steganography for secure information transmission by nanopore

Zheng-Li Hu [1†], Jia Wang[1†], Shao-Chuang Liu[1], Jia-Hong Wang[1], Hui Ma[1], Cheng Yang[3], Yi-Lun Ying[1,2], Feng Yan[3], and Yi-Tao Long[1]*

[1] Molecular Sensing and Imaging Center, School of Chemistry and Chemical Engineering, Nanjing University, Nanjing, 210023, China

[2] Chemistry and Biomedicine Innovation Center, Nanjing University; Nanjing, 210023, China

[3] School of Electronic Science and Engineering, Nanjing University, Nanjing 210023, China

[†] Equally contributed to this work.

[*] Corresponding authors: Yi-Tao Long (yitaolong@nju.edu.cn)

Methods and Experimental Section

Materials. Proaerolysin were prepared and activated by trypsin at room temperature for nanopore fabrication according to our previous works[1,2]. 1,2-diphytanoyl-sn-glycero-3-phosphocholine (DPhPC) was ordered from Avanti Polar Lipids, Inc. Potassium chloride (KCl), decane and trypsin were purchased from Sigma Aldrich Co., Ltd. Hexadecane, hexane, tris (hydroxymethyl) aminomethane (Tris), and ethylenediaminetetraacetic acid (EDTA) were from Aladdin Co., Ltd. The lipid solution was prepared by dissolving DPhPC in decane to reach a concentration of 30 mg/mL. The pretreatment solution for the Teflon testing cell was prepared by mixing hexadecane with hexane at a volume ratio of 1:99 to promote lipid bilayer formation. To minimize the effects of different translocation orientations on nanopore currents arising from structural differences between the 5’ and 3’ termini, all DNA samples (HPLC grade) were synthesized with terminal hydroxyl groups at both ends, and purified by Sangon Biotech Co., Ltd. All the DNAs was dissolved to 100 μM for further application using Tris-HCl solution that consisting of 1 mM EDTA and 10 mM Tris, pH 8.0. Unless otherwise indicated, all the solutions were prepared with ultrapure water (18.2 MΩ·cm at 25 °C) from an Arium Pro (Sartorius, USA). The nanopore experiments in this work were conducted at 22 ± 2 ℃ in Tris-KCl buffer solution that containing 1.0 M KCl, 10 mM Tris, 1.0 mM EDTA, pH 8.0.

Sample preparation for information storage. The information storage library was formed by the nonmodified poly(dA)$_3$ (A3) for address codes, and five digital photoresponsive DNAs for letter codes with the sequences of 5’-XAAAA-3’ for **M1**, 5’-AXAAA-3’ for **M2**, 5’-AAXAA-3’ for **M3**, 5’-AAAXA-3’ for **M4**, and 5’-AAAAX-3’for **M5**, respectively. Here, “X” represents the azobenzene moiety as a pseudo nucleotide attached to DNA strand through a threoninol linker, and “A” indicates the adenine deoxyribonucleotide. According to the Baudot encoding, **M1** is responsible for Bit-1, **M2** for Bit-2, **M3** for Bit-3, **M4** for Bit-4, and **M5** for Bit-5, where the absence or presence indicate the binary bit “0” or “1”, respectively. Ten different concentrations of A3 represent “0” to “9” for the order of letters in a text. An addressable letter was written into a mixture, which is composed by photoresponsive DNAs of equal concentration and A3 at a specific concentration corresponding to the letter number. A set of mixtures were premade for the ten-letter message “HELLO” “WORLD”. For example, the first letter “H” is prepared by M1 and M3 with molar concentration ratio of 1:1, and the third letter “L” is prepared by M1 and M4 of equal concentration, and A3 at a concentration of 0.2 μM. For the reading of each letter, 5 μL of sample solution was used for a nanopore measurement performed in a 500 μL home-made Teflon testing cell.

Single-molecule detection with nanopore. A Teflon sheet with an aperture of ~50 μm in diameter was fabricated to support the formation of planar lipid bilayer membrane, which separates the two chambers of a Teflon testing cell. After pretreatment of the testing cell with the hexadecane/hexane solution for ~10 minutes, each chamber was filled with 500 μL Tris-KCl buffer solution, and equipped with an Ag/AgCl electrode to apply voltages. When the lipid bilayer membrane of appropriate thickness was constructed by using the Montal-Mueller method, 1~2 μL of

the activated proaerolysin solution (0.2 mg/mL) was added into the chamber that connects to ground. The DNA samples were added into the same chamber after the insertion of a single aerolysin nanopore into the lipid bilayer membrane. Ionic currents of nanopore were recorded by an Axonpatch 200B amplifier combined with a Digidata 1550B analog-to-digital converter (Molecular Devices). Single-molecule signals of all DNAs were low-pass filtered at 5 kHz and collected at a sampling rate of 250 kHz. Note that the photoresponsive DNAs were irradiated with UV light (λ = 365 nm) for 15 minutes before the nanopore measurement (which can reduce to less than 10 minutes according to our previous studies[3,4]) to reach a photostationary state, thereby enabling high-quality and reproducibility of the experimental data. In the decoding experiment, the DNA strands were measured as a mixture. Nanopore readouts under darkness with no ambient light before (Dark) and after UV (λ = 365 nm) irradiation were acquired from at least three individual experiments. In this work, all the data used for information decoding were collected within 5 minutes after removing the UV irradiation. The ratio of the *cis*-tAzo and *trans*-tAzo isomers for **M1**-**M5** remains nearly constant when UV exposure is discontinued for 5 minutes (Figure S28), thereby enabling the precision of information encoding and decoding. Taking the letter "L" as an example, we found that all the three populations are still clearly identifiable in the 2D density scatter plots when the UV irradiation was removed for 5 minutes (Figure S29).

Decoding of the transmitted message. For each letter, nanopore sensing data were acquired to identify the coding strands for letter codes, and quantify A3 for address codes. To facilitate the identification of photoresponsive DNAs, single-molecule signals were assigned to the corresponding regions that were defined in advance: before irradiation, R1 (0.20< I/I_0 <0.50, t_D >100 ms) for **M3**, R2 (0.30< I/I_0 <0.345, 0.4 ms< t_D <100 ms) for **M2** and/or **M4**, R3 (0.345< I/I_0 <0.369, 0.4 ms< t_D <100 ms) for **M1**, and R4 (I/I_0 >0.369, 0.4 ms< t_D <100 ms) for **M5**; after UV irradiation, R1' (0.20< I/I_0<0.50, t_D >100 ms) for **M3**, R2' (0.30< I/I_0<0.334, 0.4 ms< t_D <100 ms) for **M4**, R3' (0.334< I/I_0<0.355, 0.4 ms< t_D <100 ms) for **M1** and/or **M2**, R4' (0.355< I/I_0<0.380, 0.4 ms< t_D <100 ms) for **M1**, **M4** and/or **M5**, and R5' (I/I_0>0.380, 0.4 ms< t_D <100 ms) for **M2**. We firstly estimated the concentration of A3, whose typical signals are easily recognized with I/I_0 > 0.50. The address codes can be readily determined when compared with the well-established standards of effective frequencies (f_e). We then accurately discriminated the distinct photoisomers of **M1**, **M3** and **M5** before irradiation, **M2** and **M3** after UV irradiation. Next, we continued with identifying **M2** and **M4** without illumination, **M1**, **M4** and **M5** after exposure to UV light. A collaborative analysis of the nanopore readouts with and without irradiation was performed to further identify the 5 coding DNAs, especially for **M2** and **M4**. Subsequently, we analyzed the confusing results and extracted the common outputs under two different light irradiation situations, thus decoding every letter back using the Baudot encoding scheme. Full recovery of the original message can be achieved in a few minutes by the intended receiver.

As the coding DNAs are presynthesized, the readout time per cycle mainly consists of three parts, and can be calculated by t_{read} = $t_{irradiation}$ + $t_{detection}$ + t_{data}, where $t_{irradiation}$ represents light irradiation time of sample, $t_{detection}$

represents DNA detection time by nanopore, and t_{data} is data processing and analysis time. Taking the letter "L" as an example, firstly, the sample was irradiated with UV (λ = 365 nm) light for 15 minutes to reach a photostationary state, which can be reduced to 5 minutes (see Fig. S14); After that, the sample was added into the testing cell, and data were recorded with aerolysin nanopore for 5 minutes, resulting in a recognizable 2D density scatter plot based on single-molecule signals collected even in a period of 1 minute (see Figs. S15-S18); Next, data processing and analysis would be completed within 1 minute by using the customized python-based software programs. Therefore, the readout time is calculated to be 8 minutes in an optimized writing/reading cycle.

Data processing and analysis. Each point in the scatter plots represents a single-molecule blockade event. Single molecular signals with a current threshold ($>2\times I_{RMS}$) were extracted and analyzed using PyNanoLab software. The collision events with duration times shorter than 0.4 ms were excluded for the statistical analysis. Unless otherwise stated, the translocation events with t_D > 0.4 ms and I/I_0 > 0.50 were collected for the quantitative analysis of A3. The average values of I/I_0 were established by the Gaussian fits of current histograms, and lg(t_D) were determined by the Gaussian fitting to the transformed duration histograms. The effective frequencies (f_e) were calculated by $f_e = N / t_e$ according to our previous study[5], where N is the accumulated number of typical signals, the effective time $t_e = t_{total} - t_{off}$, t_{total} is the recording time, and t_{off} is the cumulative blocked time when other samples are occupying the nanopore. For the DNA mixtures, the residual currents of single molecular signals were calibrated by using A3 as an internal reference. Plotted values are shown as mean ± s. d., where the mean values were obtained from at least three independent nanopore experiments. The error bars represent the standard deviation of the mean fitted values (for example, I/I_0 and lg(t_D)) or the mean calculated values (for example, f_e) from three independent experiments.

Letter ►	A	B	C	D	E	F	G	H	I	J	K	L	M	N	O	P	Q	R	S	T	U	V	W	X	Y	Z
Bit-1 ►	0	1	0	0	0	0	1	1	0	0	0	1	1	0	1	1	1	0	0	1	0	1	1	1	1	1
Bit-2 ►	0	1	1	1	0	1	1	0	0	1	1	0	1	1	1	0	0	1	0	0	0	1	0	1	0	0
Bit-3 ►	0	0	1	0	0	1	0	1	1	0	1	0	1	1	0	1	1	0	1	0	1	1	0	1	1	0
Bit-4 ►	1	0	1	0	0	0	1	0	1	1	1	1	0	0	0	1	1	1	0	0	1	1	1	0	0	0
Bit-5 ►	1	1	0	1	1	1	0	0	0	1	1	0	0	0	0	0	1	0	1	0	1	0	1	1	1	1
**M1** ►	-	+	-	-	-	-	+	+	-	-	-	+	+	-	+	+	+	-	-	+	-	+	+	+	+	+
**M2** ►	-	+	+	+	-	+	+	-	-	+	+	-	+	+	+	-	-	+	-	-	-	+	-	+	-	-
**M3** ►	-	-	+	-	-	+	-	+	+	-	+	-	+	+	-	+	+	-	+	-	+	+	-	+	+	-
**M4** ►	+	-	+	-	-	-	+	-	+	+	+	+	-	-	-	+	+	+	-	-	+	+	+	-	-	-
**M5** ►	+	+	-	+	+	+	-	-	-	+	+	-	-	-	-	-	+	-	+	-	+	-	+	+	+	+

Figure S1. Baudot codes of the 26 English letters corresponding to the five digital coding DNAs. The absence or presence of coding strands represent bit "0" or "1", respectively.

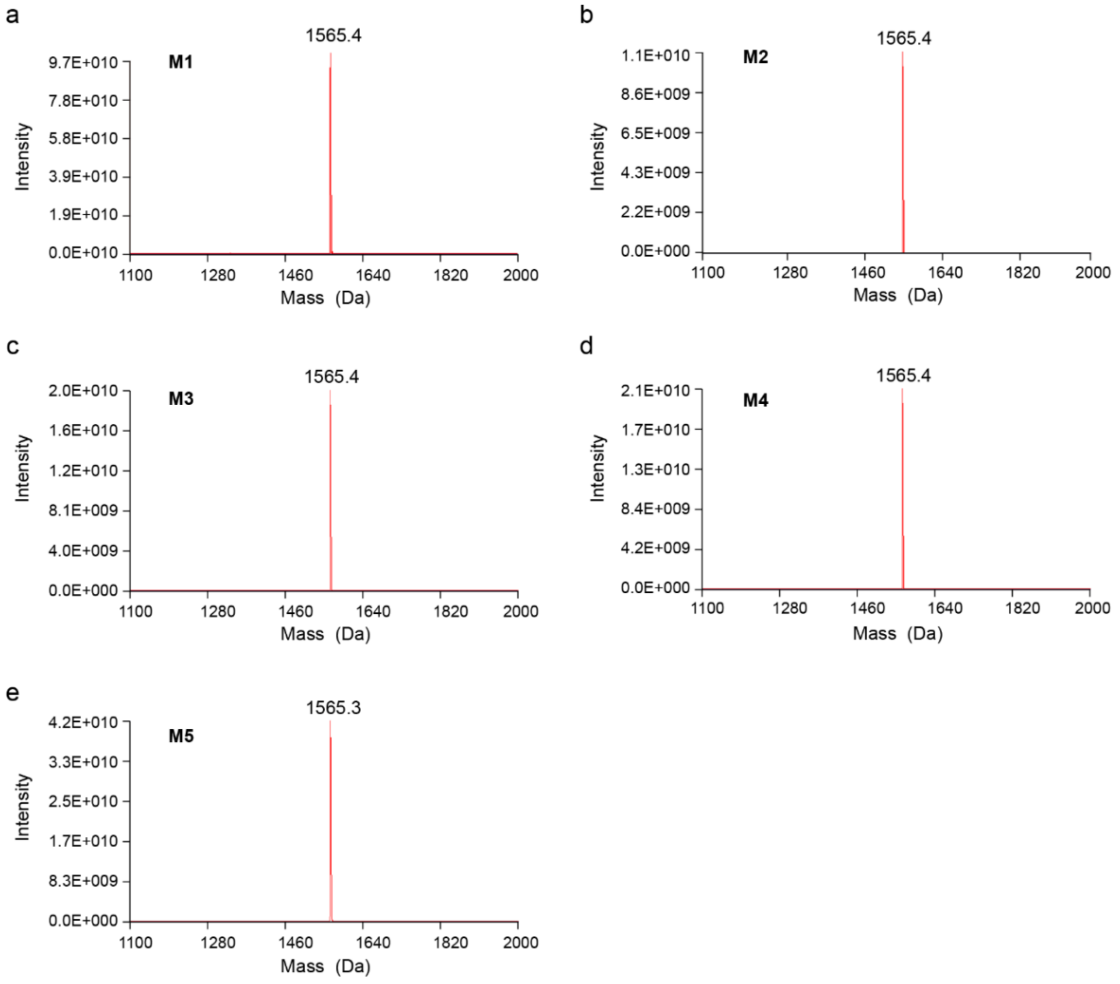

Figure S2. The Mass spectra of (a) **M1**(XA4), (b) **M2** (AXA3), (c) **M3** (A2XA2), (d) **M4** (A3XA), and (e) **M5** (A4X), respectively. The heterogeneous signal intensities arise from variations in initial sample concentrations during synthesis and subsequent mass spectrometry characterization. Data were obtained from Sangon Biotech Co., Ltd.

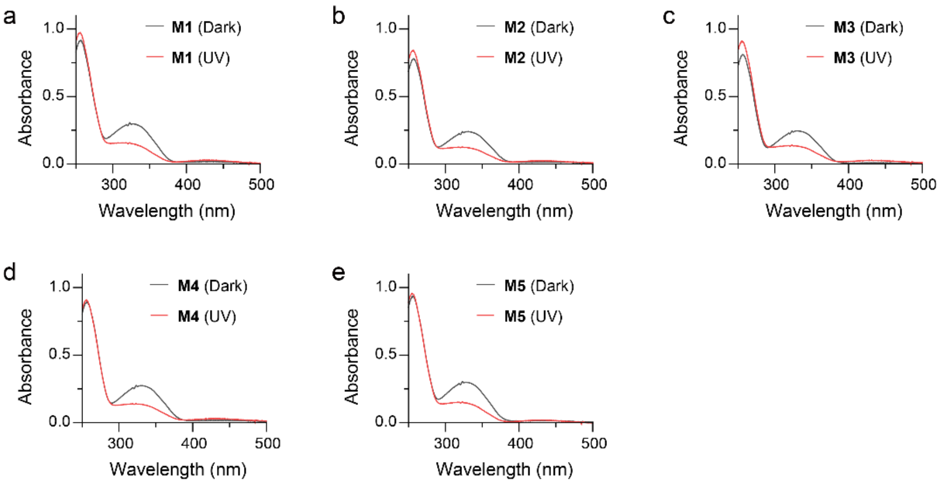

Figure S3. UV-Vis spectra of (**a**) **M1**(XA4), (**b**) **M2** (AXA3), (**c**) **M3** (A2XA2), (**d**) **M4** (A3XA), and (**e**) **M5** (A4X) before and after UV irradiation. The final concentration of each photoresponsive DNA was fixed at 10.0 μM.

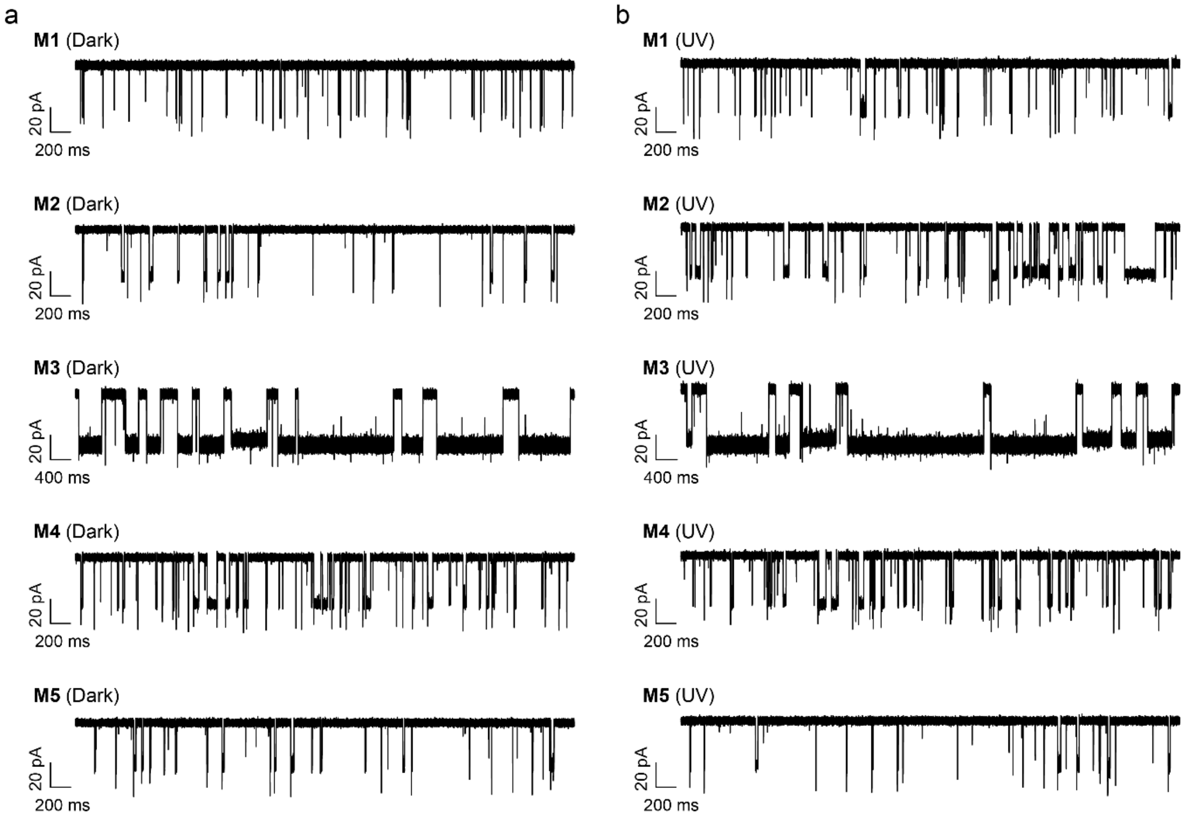

Figure S4. Real-time current traces of **M1**(XA4), **M2** (AXA3), **M3** (A2XA2), **M4** (A3XA), and **M5** (A4X) before (**a**) and after UV irradiation (**b**), from top to bottom. The five informational DNAs were detected by aerolysin nanopore at +120 mV.

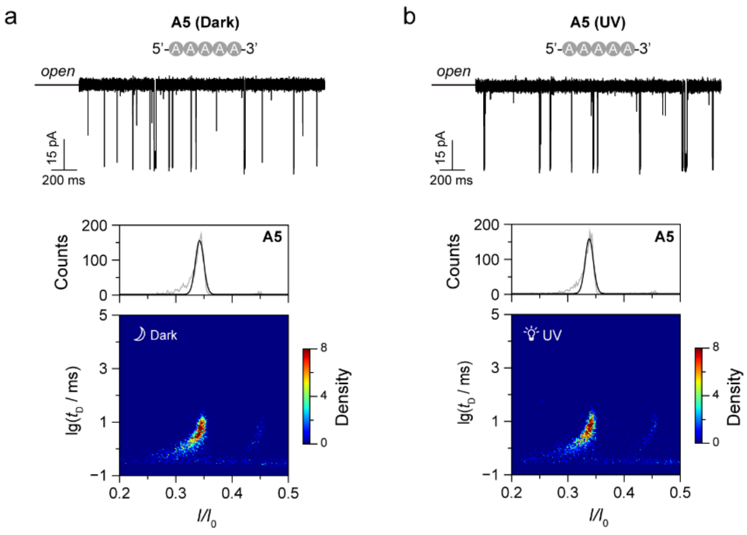

Figure S5. Raw current traces, current histograms and 2D density scatter plots of I/I_0 vs. lg(t_D) for the unmodified poly(dA)$_5$ (A5) before (a) and after (b) UV irradiation. Data were obtained at the voltage of +120 mV.

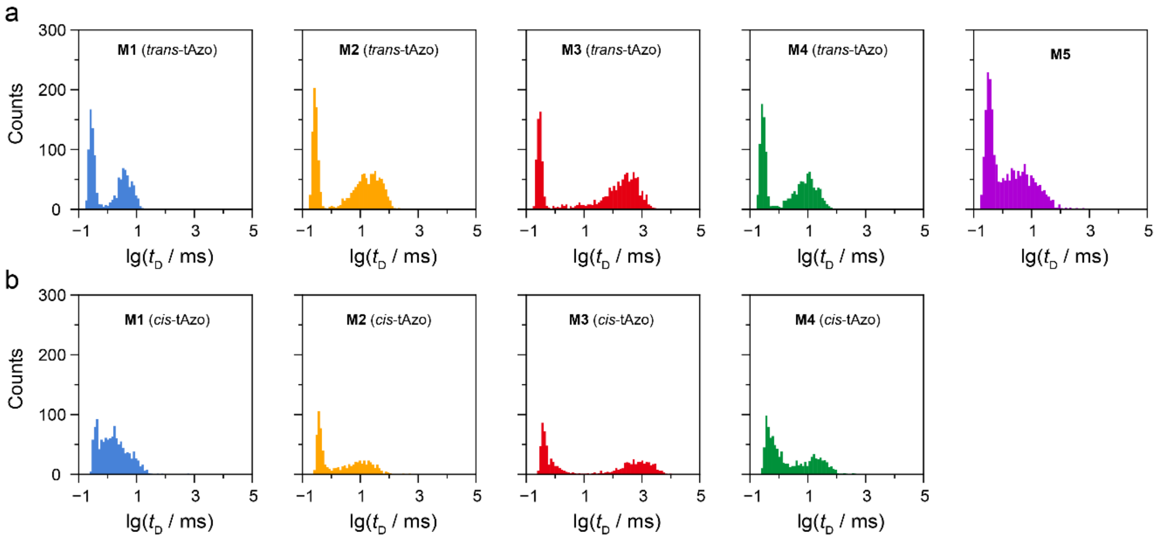

Figure S6. Duration histograms of the five informational DNAs for their *trans*-tAzo (**a**) and *cis*-tAzo isomers (**b**). For **M5**, the *trans*-tAzo and *cis*-tAzo conformations are indistinguishable either by current blockage or duration time. Data were obtained after UV irradiation at the voltage of +120 mV.

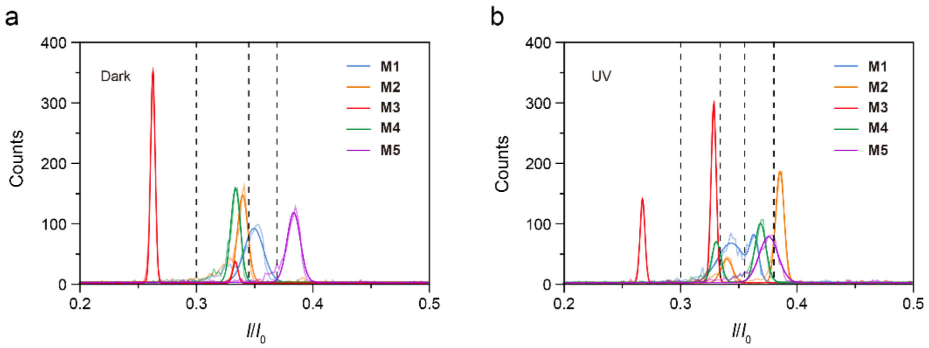

Figure S7. Current distributions of the five digital DNAs before (**a**) and after (**b**) UV irradiation. Dash lines indicate the thresholds of I/I_0 for molecular identification. Current separation between two peaks was determinded by $S = 2\times|I_2/I_0 - I_1/I_0| / |W_2+W_1|$, where I_1/I_0 and I_2/I_0 are the mean residual current blocakge for Peak 1 and Peak 2, W_1 and W_2 denote their corresponding peak widths[4]. Before irradiation, *trans* conformers dominate, allowing direct identification of **M1** and **M5**, while **M2**/**M4** (S=0.35) remain indistinguishable. After irradiation, a large fraction of molecules undergoes *trans*-to-*cis* isomerization, eanbling **M2**(*cis*) and **M4**(*trans*) to be resolved, whereas **M2**(*trans*)/**M1**(*trans*) (S=0.12) and **M1**(*cis*)/**M4**(*cis*)/**M5** ($S\leqslant$0.31) remain ambiguous.

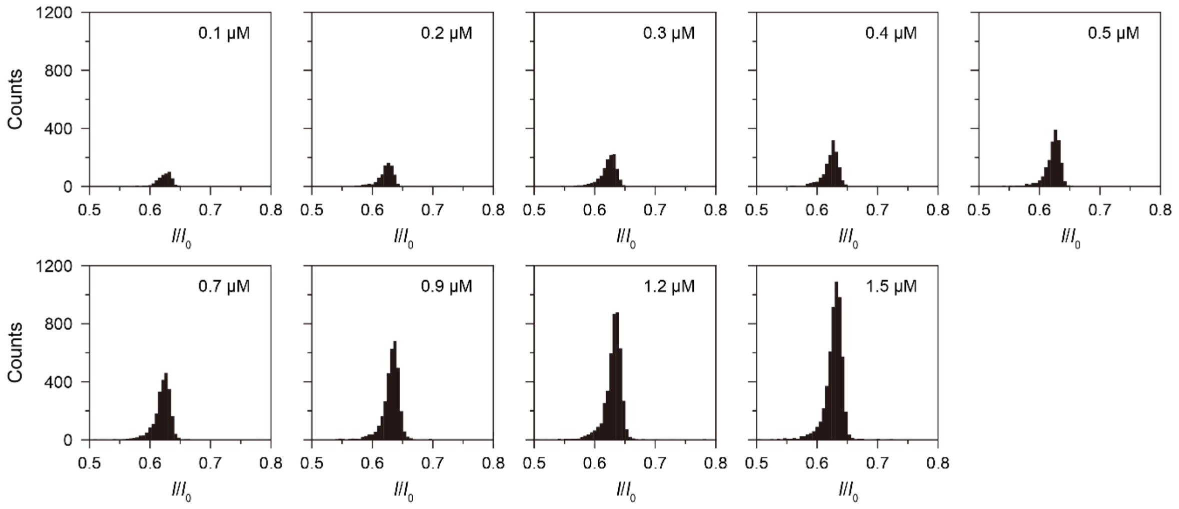

Figure S8. Current histograms of A3 at different concentrations varying from 0.1 to 1.5 μM. The collision events with duration times shorter than 0.4 ms were excluded for statistical analysis to minimize the evaluation errors. Data were collected from pure samples by aerolysin nanopore during single-channel recording of 5 minutes at +120 mV.

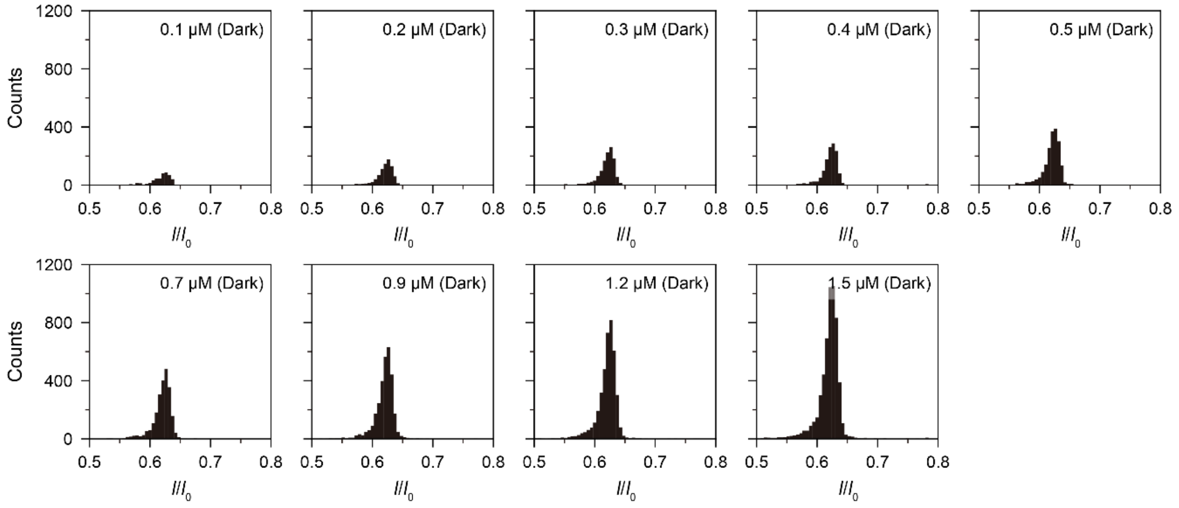

Figure S9. Current histograms of A3 at concentrations increasing from 0.1 to 1.5 μM. The collision events with duration times shorter than 0.4 ms were excluded for statistical analysis. Data were obtained from mixtures for the ten-letter message before UV irradiation during single-channel recording of 5 minutes at +120 mV.

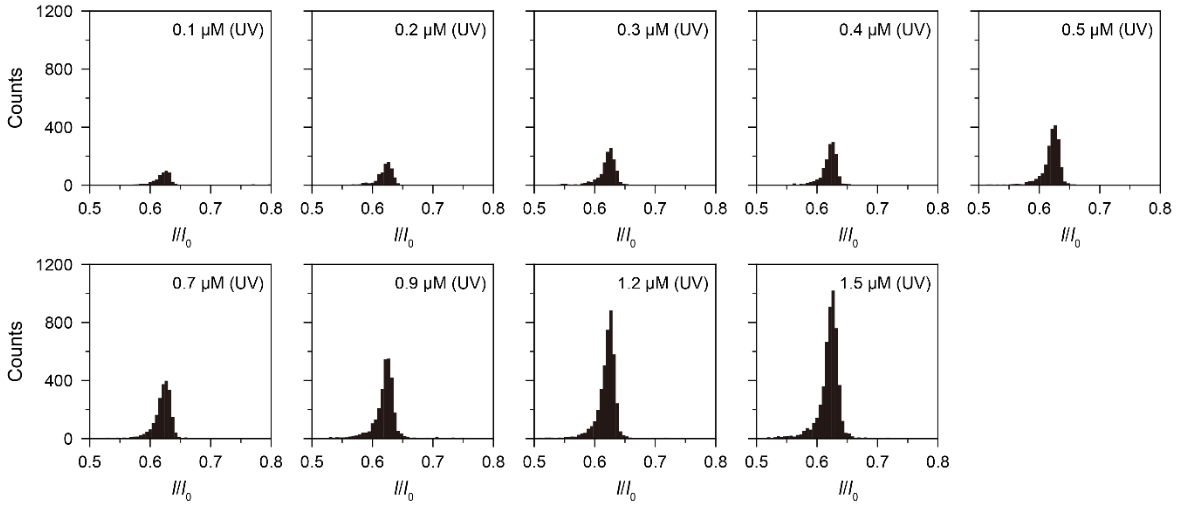

Figure S10. Current histograms of A3 at concentrations improving from 0.1 to 1.5 μM. The collision events with duration times shorter than 0.4 ms were excluded for statistical analysis. Data were obtained from mixtures for the ten-letter message after UV irradiation during single-channel recording of 5 minutes at +120 mV.

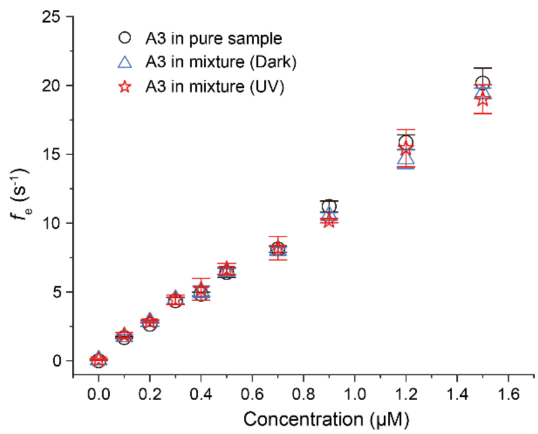

Figure S11. The effective frequencies (f_e) of A3 dependent on concentrations in pure and mixture samples. The average values of f_e increase linearly with the concentrations of A3 improving either in pure sample or mixtures. Error bars were estimated from three individual nanopore experiments.

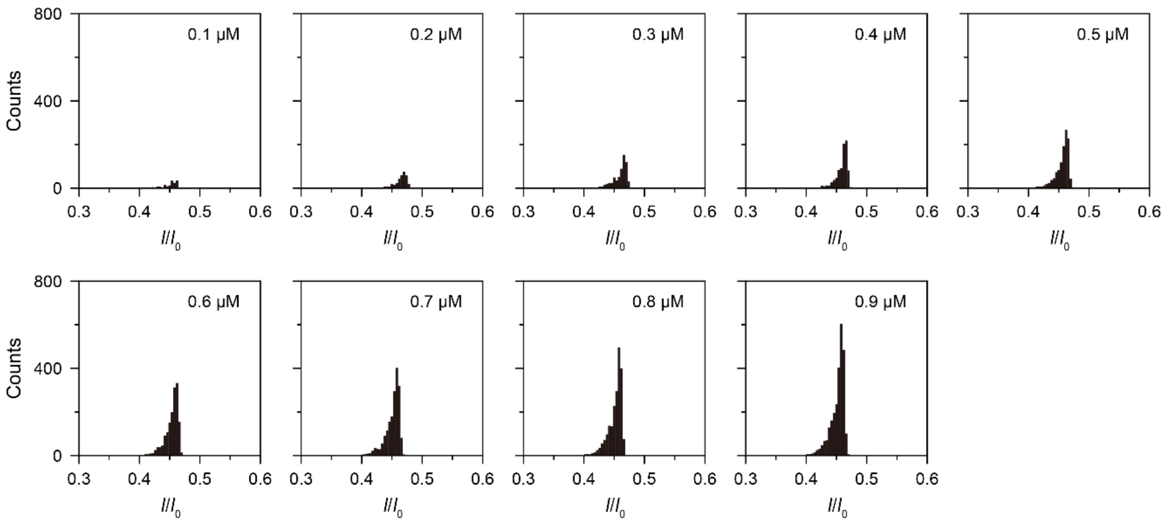

Figure S12. Current histograms of poly(dA)$_4$ (A4) at different concentrations varying from 0.1 to 0.9 μM. The collision events with duration times shorter than 0.4 ms were excluded for statistical analysis to minimize the evaluation errors. Data were collected from pure samples by aerolysin nanopore during single-channel recording of 5 minutes at +120 mV.

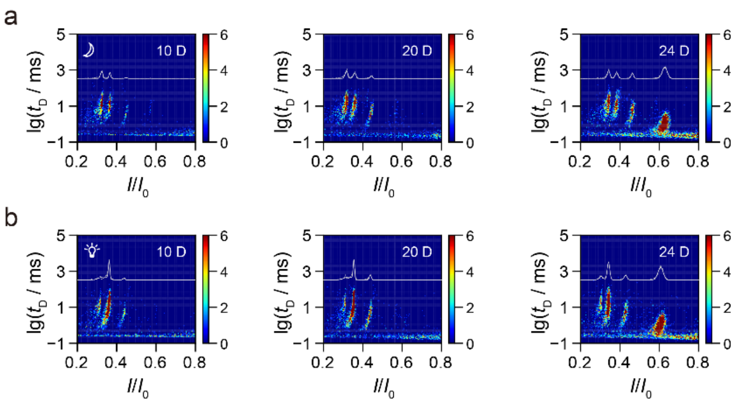

Figure S13. 2D density scatter plots for the addressable character "10 D", "20 D" and "24 D" before (**a**) and after UV irradiation (**b**), from left to right. The system employs two molecular species, A3 for the units place and A4 for the tens place, each with ten concentration gradients representing address codes from 0 to 9. Data were acquired from the corresponding current traces at +120 mV by using aerolysin nanopore.

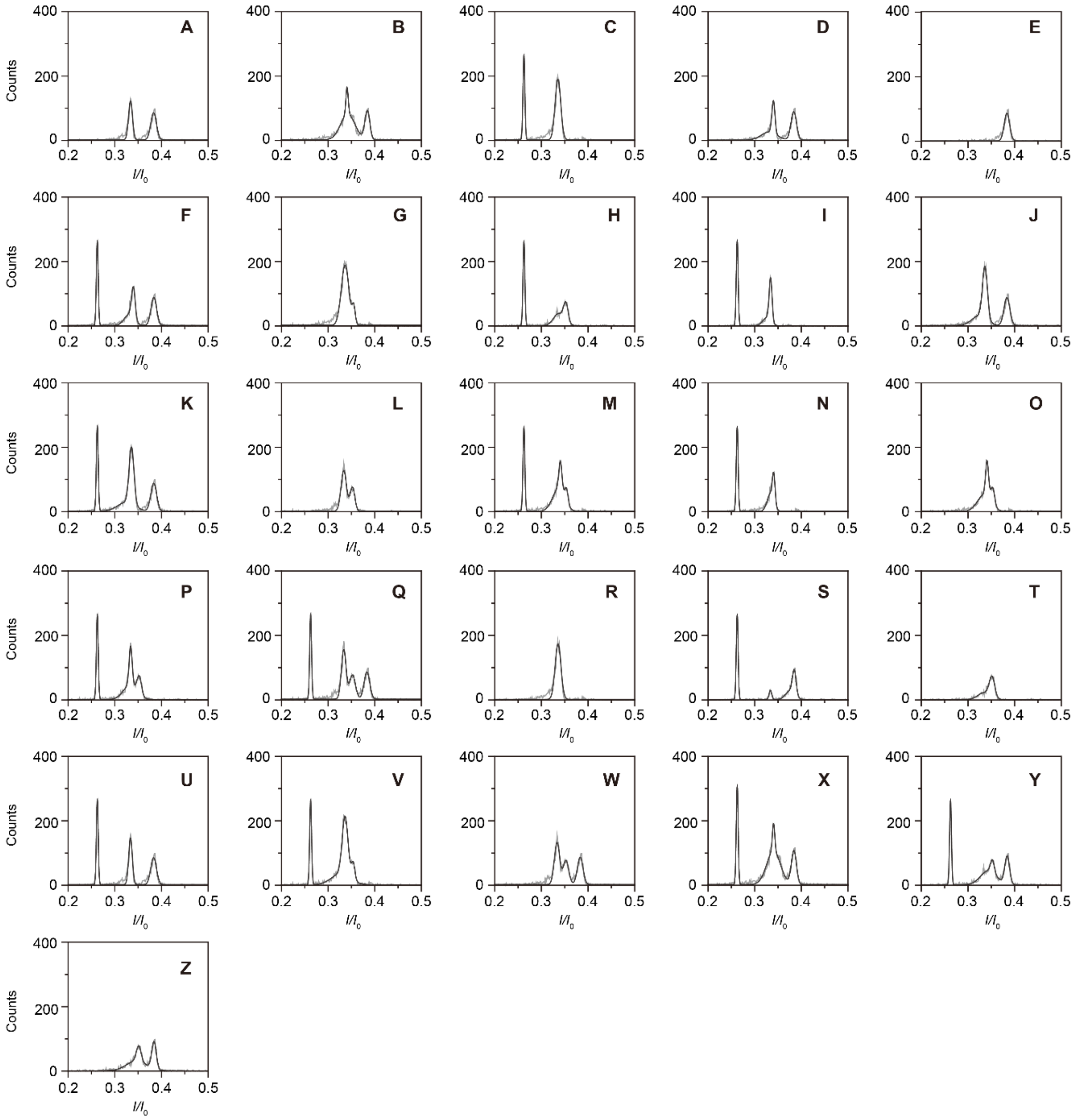

Figure S14. Current distributions for 26 English letters before UV irradiation. Each plot was produced by the premixed signals of pure DNA samples. Data were obtained at the voltage of +120 mV.

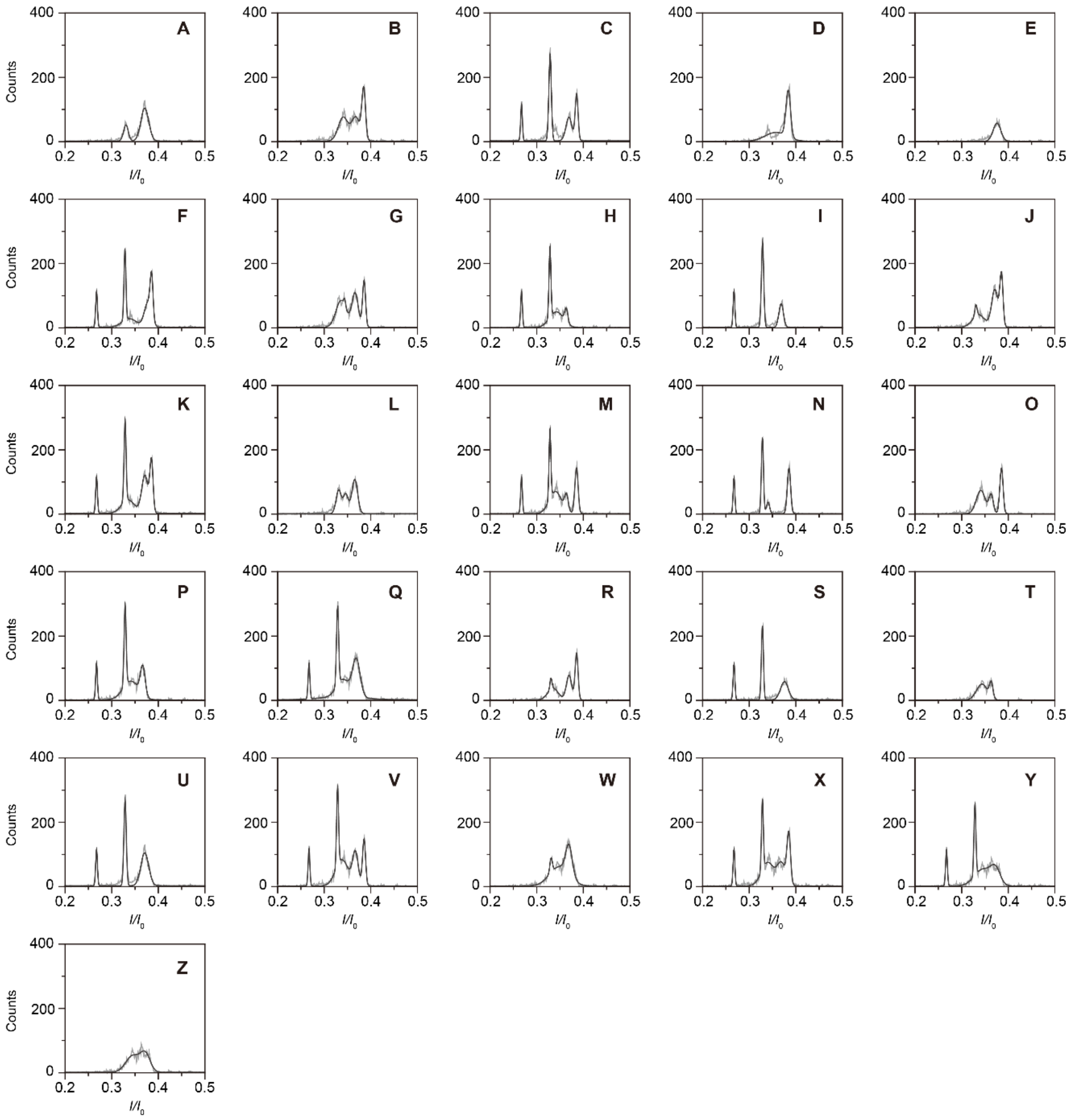

Figure S15. Current distributions for 26 English letters after UV irradiation. Each plot was produced by the premixed signals of pure DNA samples. Data were obtained at the voltage of +120 mV.

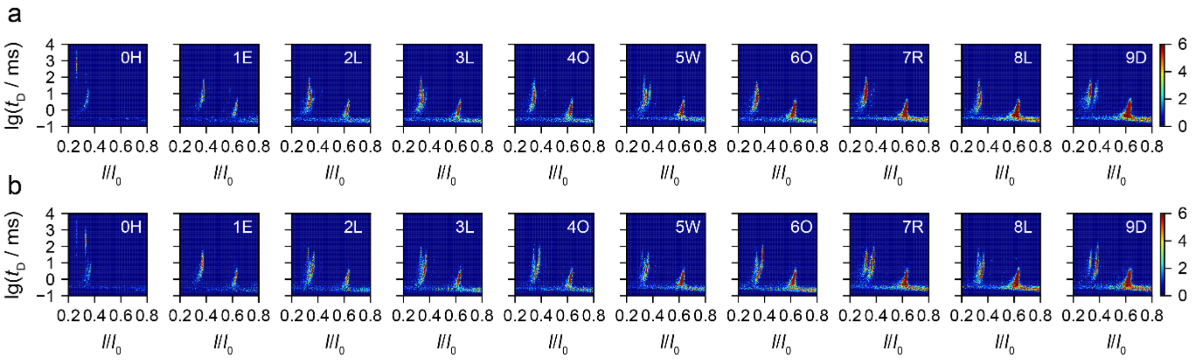

Figure S16. 2D density scatter plots for the addressable ten-letter message before (**a**) and after UV irradiation (**b**). Data were acquired from the corresponding current traces at +120 mV by using aerolysin nanopore.

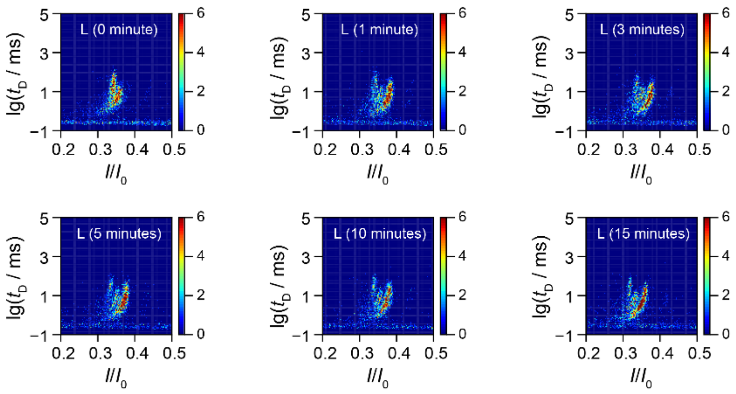

Figure S17. 2D density scatter plots for the character “L” at different UV light irradiaiton time from 0-15 minutes. Data were acquired from the corresponding current traces after UV (λ =365 nm) irradiation at +120 mV by using aerolysin nanopore.

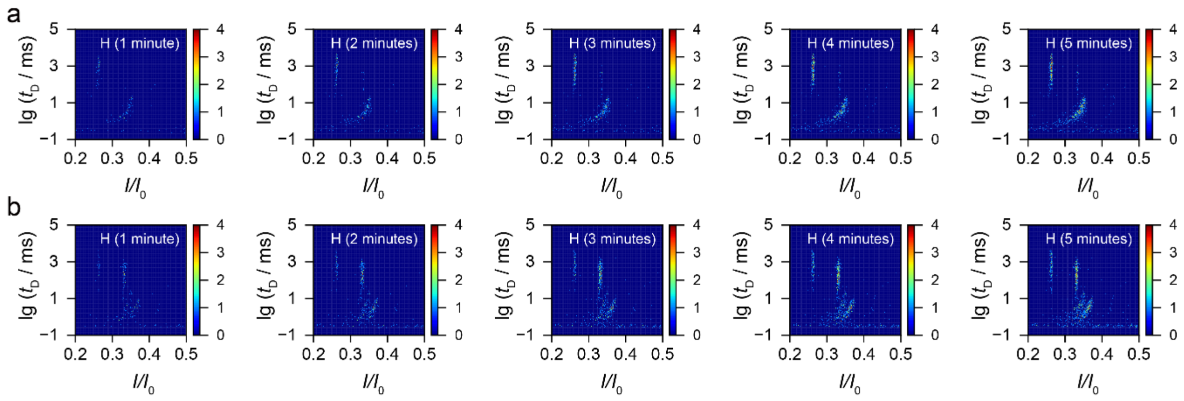

Figure S18. 2D density scatter plots for the letter "H" before (**a**) and after UV irradiation (**b**). Data were acquired at +120 mV from the current traces of 1 minute, 2 minutes, 3 minutes, 4 minutes and 5 minutes, respectively (from left to right). The populations for coding strands are recognizable even in the 1 minute-scatter plot.

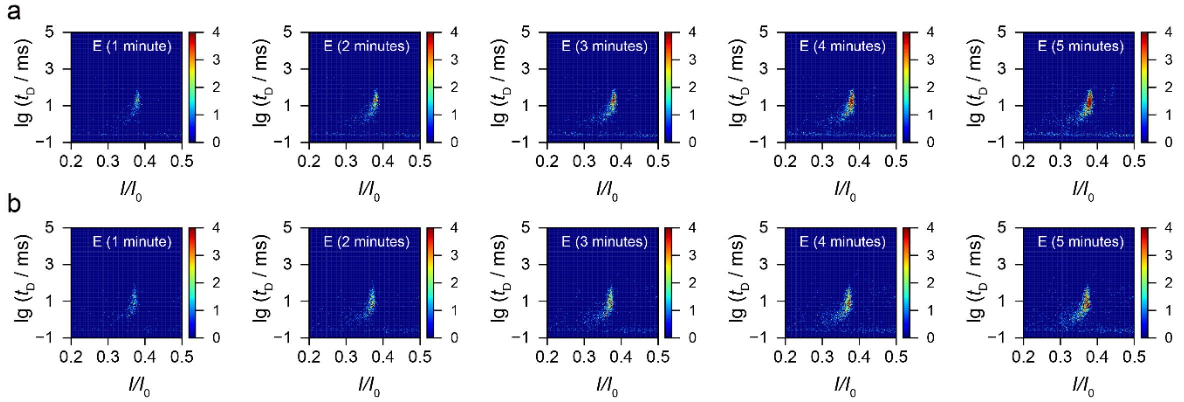

Figure S19. 2D density scatter plots for the letter "E" before (**a**) and after UV irradiation (**b**). Data were acquired at + 120 mV from the current traces of 1 minute, 2 minutes, 3 minutes, 4 minutes and 5 minutes, respectively (from left to right). The populations for coding strands can be clearly recognized in the 1 minute-scatter plot.

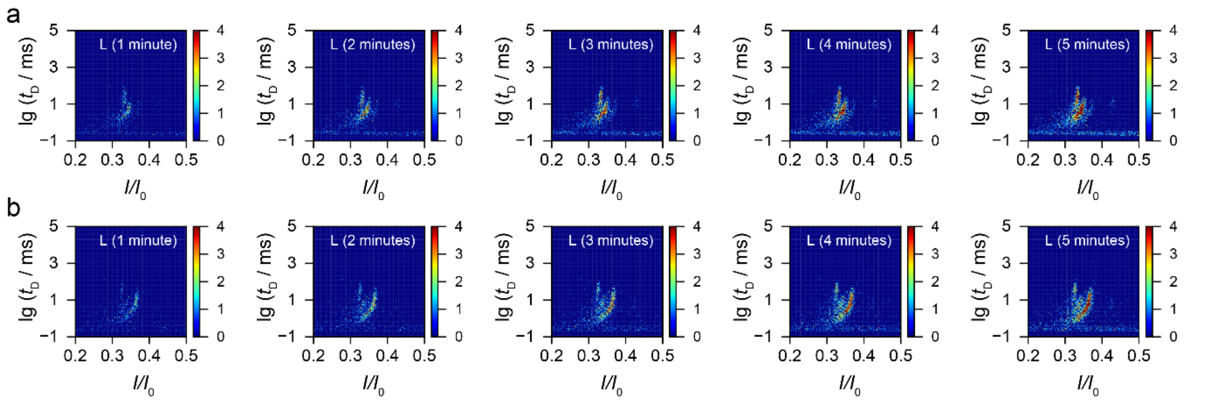

Figure S20. 2D density scatter plots for the letter "L" before (**a**) and after UV irradiation (**b**). Data were acquired at + 120 mV from the current traces of 1 minute, 2 minutes, 3 minutes, 4 minutes and 5 minutes, respectively (from left to right). The populations for coding strands are distinguishable in the 1 minute-scatter plot.

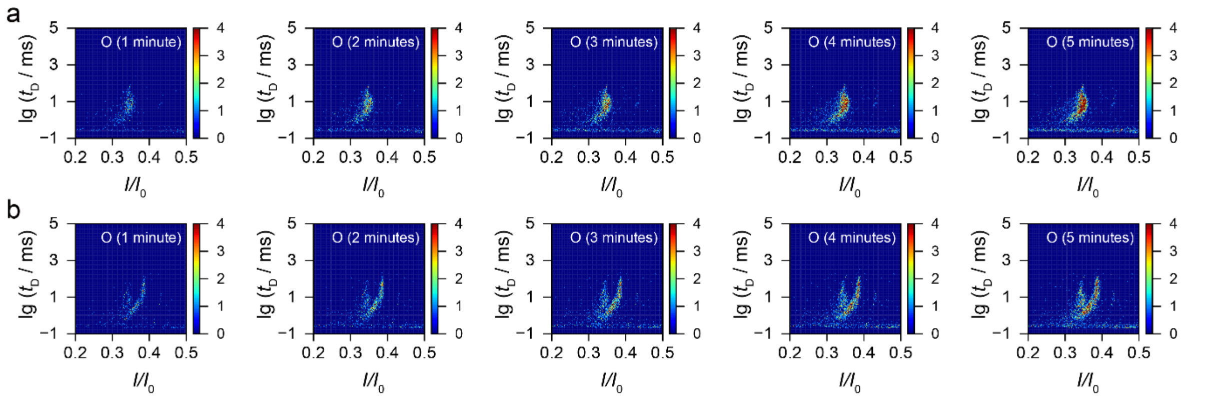

Figure S21. 2D density scatter plots for the letter "O" before (**a**) and after UV irradiation (**b**). Data were acquired at + 120 mV from the current traces of 1 minute, 2 minutes, 3 minutes, 4 minutes and 5 minutes, respectively (from left to right). The distinct populations for coding strands can be identified in the 1 minute-scatter plot.

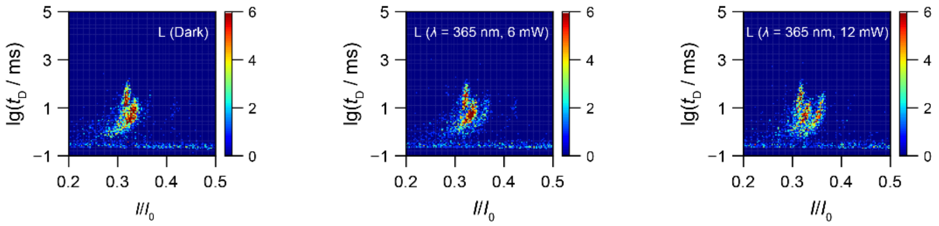

Figure S22. 2D density scatter plots for the character "L" before (Dark) and after UV irradiation of different light intensity (λ = 365 nm, 6 mW or 12 mW). Data were acquired from the corresponding current traces before or after irradiation of 15 minutes at + 120 mV by using aerolysin nanopore.

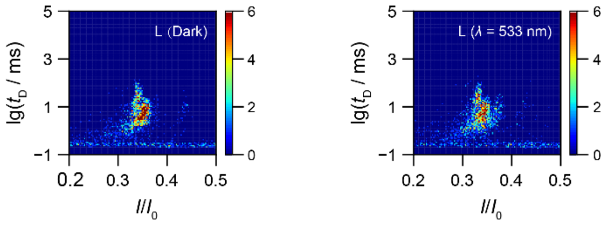

Figure S23. 2D density scatter plots for the character "L" before (Dark) and after visible light (λ = 533 nm) irradiation of 15 minutes (**b**). Data were acquired from the corresponding current traces at + 120 mV by using aerolysin nanopore.

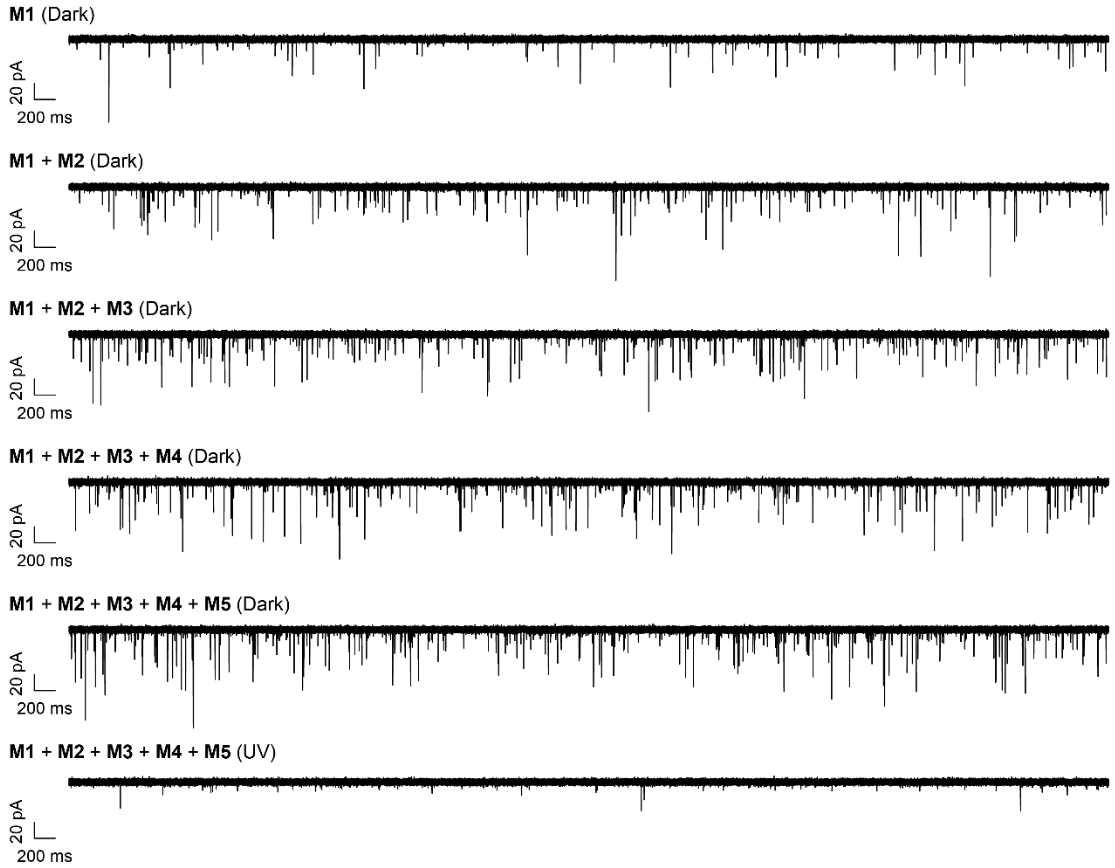

Figure S24. Real-time current traces of the coding DNAs and mixtures before and after UV irradiation. The equal-molar **M1**-**M5** were sequentially added into the *cis* chamber, with the mixture at a final concentration of 2.0 μM. Data were acquired at +120 mV by using α-hemolysin nanopore.

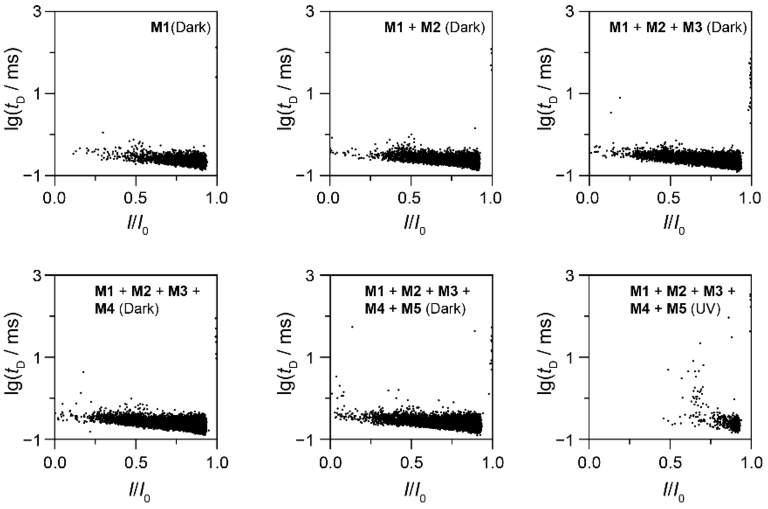

Figure S25. Scatter plots for the coding DNAs and mixtures before and after UV irradiation. Data were collected from the corresponding current traces at +120 mV by using α-hemolysin nanopore.

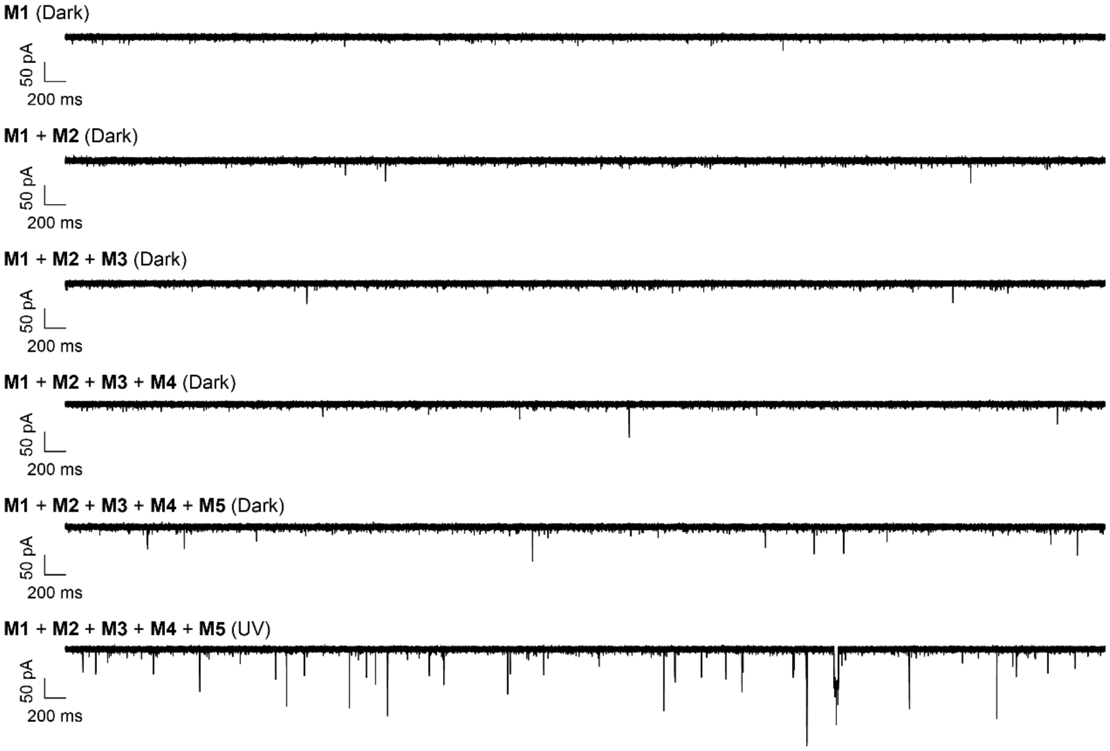

Figure S26. Real-time current traces of the coding DNAs and mixtures before and after UV irradiation. The equal-molar **M1**-**M5** were sequentially added into the *cis* chamber, with the mixture at a final concentration of 2.0 μM. Data were acquired at +120 mV by using MspA nanopore.

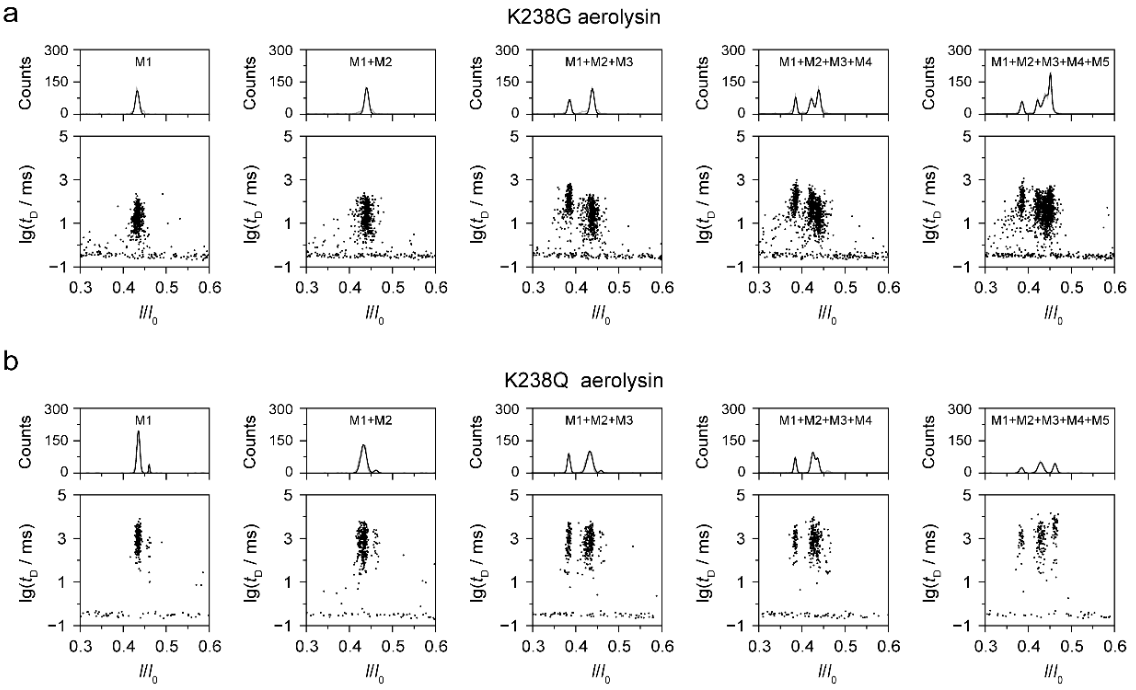

Figure S27. Current histograms and scatter plots of I/I_0 vs. lg(t_D) for the coding DNAs and mixtures detected by K238G mutant aerolysin (**a**) and K238Q mutant aerolysin nanopore (**b**), respectively. The equal molar **M1**-**M5** were sequentially added into the *cis* chamber, with the mixture at a final concentration of 0.6 μM. Data were acquired before UV irradiation at the voltage of +120 mV.

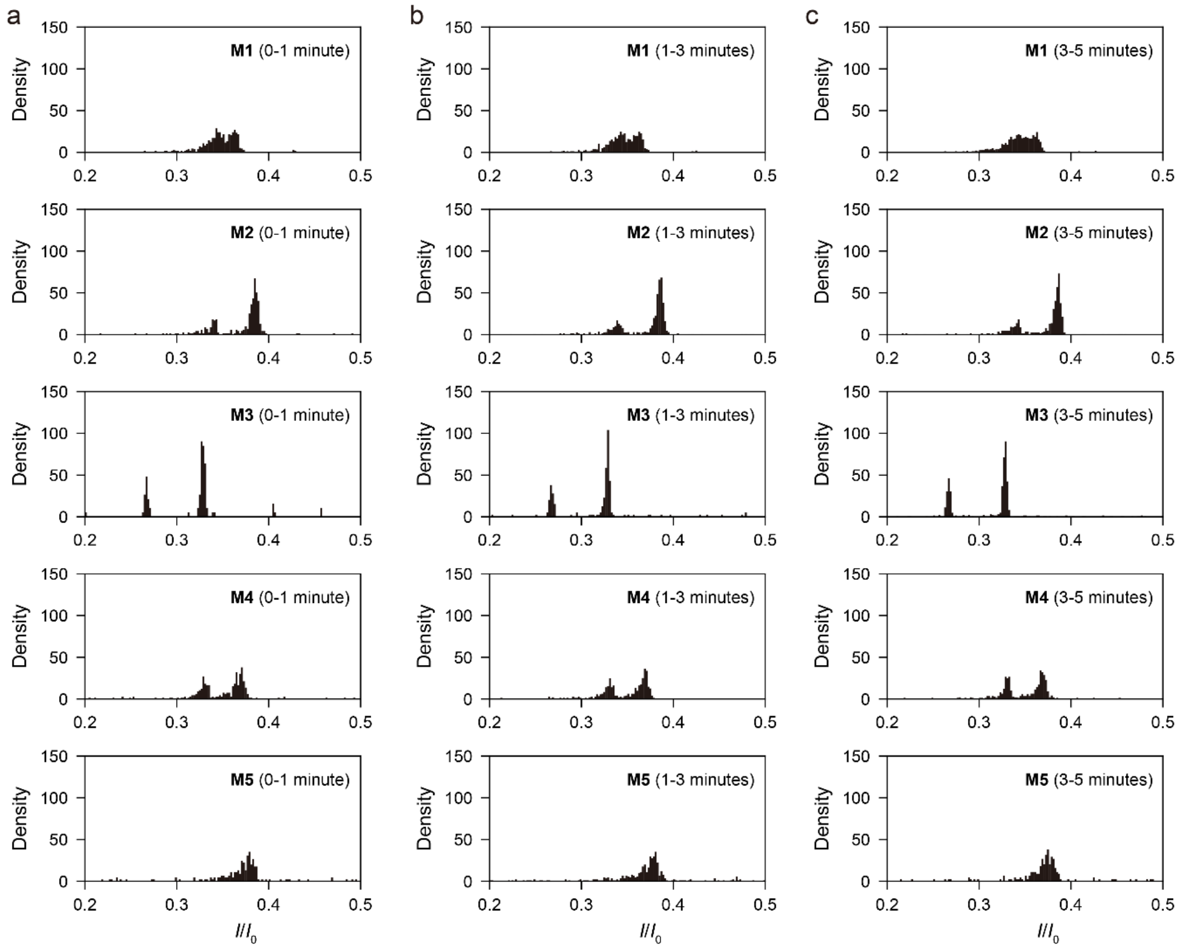

Figure S28. Current histograms of **M1**-**M5** measured during the time intervals 0-1 minute (**a**), 1-3 minutes (**b**), and 3-5 minutes (**c**) after removing the UV light. The final concentration of **M1**-**M5** is fixed at 1.0 μM. Data were collected at +120 mV by using aerolysin nanopore.

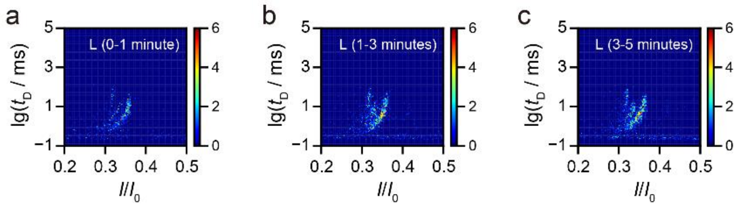

Figure S29. 2D density scatter plots of the character "L" measured during the time intervals 0-1 minute (a), 1-3 minutes (b), and 3-5 minutes (c) after removing the UV light. Data were collected from the corresponding current traces at +120 mV by using aerolysin nanopore.

Table S1. List of alternative methods for molecular information storage and encryption.

Researches*	De nove/ Synthesis-free writing	Molecules required for information storage	Logical storage density	Reading technique	Readout time per circle including sample processing	Bit error rate	Information encryption
This work (NAPDISS)	Synthesis-free	5-nt coding DNA strands	0.2-1.0 bits/nt	Biological nanopore sensing	<10 minutes	Recover all information without any error	Yes
DNA Fountain[6]	De novo	200-nt coding DNA strands	1.57 bits/nt	Illumina sequencing	>300 minutes	Recover all information without any error	No
High-density random-access storage[7]	De novo	150-nt coding DNA strands	1.1 bits/nt	Illumina sequencing	>300 minutes	Recover all information without any error	No
High-density random-access storage[7]	De novo	110-nt coding DNA strands	1.1 bits/nt	Nanopore sequencing	>60 minutes	11.4%-13.2%	No
DNA origami cryptography[8]	Synthesis-free	43-51-nt biotinylated message strands, 20-56-nt staples, 7249-nt DNA scaffold	< 0.023 bits/nt	Atomic force microscopy (AFM) imaging	60-120 minutes	0%-42.4%	Yes
DNA nanostructure-based hard drives[9]	Synthesis-free	34-nt biotinylated oligos, 70-nt DNA overhangs, 7228-nt DNA scaffold	< 0.014 bits/nt	Solid-state nanopore sensing	~60 minutes	4.50%-33.3%	Yes
Chemical-based digital polymers[10]	De novo	8-mer oligo(phos-phodiester)s	1.0 bit/mer	Tandem mass spectrometry (MS/MS) sequencing	>60 minutes	Recover all information without any error	Yes

*Comparison of NAPDISS with representative state-of-the-art (SOTA) methods was conducted using the following essential metrics:

Synthesis complexity is evaluated based on two sub-criteria: (i) whether *de novo* or synthesis-free writing approaches were employed, and (ii) the types of molecules or materials required to store the target information.

NAPDISS, DNA origami cryptography[8] and DNA nanostructure-based hard drives[9] are structure-based information storage approaches that employ pre-synthesized DNAs and are thus classified as synthesis-free writing. In comparison, DNA Fountain[6], high-density random-access storage[7] and chemical-based digital polymers[10]

are sequence-based information storage schemes relying on *de novo* synthesis of additional unique DNA sequences or polymer chains to store new data.

In our NAPDISS system, only 5-nt photoresponsive DNAs need to be synthesized, and these are reusable. In contrast, DNA origami cryptography requires 43-51-nt biotinylated message strands, 20-56-nt staples, and 7,249-nt DNA scaffold to form correctly folded origamis recognizable by AFM imaging[8]. DNA nanostructure-based hard drives require 34-nt biotinylated oligonucleotides, 70-nt overhangs, and 7,228-nt DNA scaffold to assemble nanostructures distinguishable via solid-state nanopore sensing[9]. DNA Fountain[6] and high-density random-access storage[7] typically use 100-200-nt DNA sequences. Chemical-based digital polymers store information in synthetic polymer-monomer sequences, with decoding typically performed by mass spectrometry; in the example shown in this table, 8-mer oligo(phosphodiester) constructs are used as the storage medium[10].

Logical storage density (bits/nt) indicates the theoretical limit of information that can be stored or encoded, in bits, divided by the number of coding DNA nucleotides.

NAPDISS utilizes modular 5-nt DNA sequences for information storage, encoding 5 bits of data in 5-25 nucleotides, yielding a logical storage density of 0.2-1.0 bits/nt.

DNA Fountain has a reported net information density of 1.57bits/nt[6], and high-density random-access storage achieves 1.1bits/nt[7], both excluding primers.

DNA origami cryptography[8] employs biotinylated message strands (43-51 nt), staple strands (20-56 nt), and a 7249-nt DNA scaffold that self-assembles into braille-like patterns for information storage. Each spot represents 1 bit of binary data through the combination of the scaffold and a message strand within the origami structure, with proper folding facilitated by the corresponding staple strands. Based on a 43-nt message strand, the storage density is calculated to be less than 1 bit per 43 nt (approximately 0.023 bits/nt).

DNA nanostructure-based hard drives[9] use 34-nt biotinylated oligos (complementary to the ssDNA overhang), 70-nt ssDNA overhangs, and a 7228-nt ssDNA scaffold to encode binary data, with overhang-scaffold binding representing “0” and biotinylated oligo-overhang-scaffold complexes representing “1” (with bound streptavidin). Based on the overhang length, the storage density is less than 1 bit per 70 nt (approximately 0.014 bits/nt).

Chemical-based digital polymers[10] utilize sequence-coded poly(phosphodiester)s comprising eight monomer units (for example, light-cleavable o-nitrobenzyl ether and stable p-nitrobenzyl ether motifs) to store 8 bits of binary information, thereby achieving a storage density of 1 bit per monomer.

Readout/Decryption time per circle primarily consists of the time required for sample processing, sensing or sequencing, data analysis, and decoding.

NAPDISS requires only a few minutes of light exposure for pre-synthesized sample processing and about 2 minutes of nanopore detection to obtain nanopore current spectra for decoding, thereby enabling information decryption within 10 minutes (see Methods and Experimental Section).

DNA Fountain[6] and high-density random-access DNA storage[7] require PCR amplification of the encoding sequences, followed by decoding through Illumina or nanopore sequencing; the entire process typically requires several hours in total.

DNA origami cryptography[8] and DNA nanostructure-based hard drives[9] rely on the hybridization of complementary sequences and streptavidin binding to form detectable DNA origamis or nanostructures, which are decoded by AFM imaing or solid-state nanopore sensing. The entire process, including sample processing, usually takes 1-2 hours.

Chemical-based digital polymers[10] require UV irradiation of the oligo(phosphodiester) for about 60 minutes to

recover the modified monomer sequences, which are subsequently decoded through MS/MS sequencing; therefore, the entire process takes more than 1 hour.

Bit error rate represents the ratio of incorrectly identified bits to the total number of stored information bits.

NAPDISS employs biological nanopore sensing to identify photoresponsive DNAs and decode the encoded information, which in principle enables full recovery of the stored data.

DNA Fountain[6] and high-density random-access DNA storage[7] using Illumina sequencing and error-correcting algorithms achieve error-free information recovery. High-density random-access DNA storage[7] based on nanopore sequencing showed coordinate error rates of 11.4% and 13.2% for file A and B, respectively.

DNA origami cryptography[8] was decoded via AFM imaging, yielding individual bit-error probabilities of 42.4 %, 27.7 %, 34.7 %, 32.2 %, 39.3 %, 20.6 %, 32.7 %, and 25.2 % for the message "19120623". Full recovery of the encoded information was achieved through multiple scans. For example, in the case of a 256-pixel panda image, the error rate decreased from 16.7 % after 5 scans to 0 % after 70 scans. Similarly, for a 48-note musical sequence, the error rate dropped from 16.1 % after 5 scans to 0 % after 48 scans, enabling error-free reconstruction after 48 scans.

DNA nanostructure-based hard drives[9] were decoded using solid-state nanopore sensing. For the blank control corresponding to bit "0," the bit-error-rate values from three independent tests were 4.50 %, 4.76 %, and 12.0 %. In the first writing-reading cycle for bit "1" (Table S1, Supplemental Material), the bit-error-rate values from three tests were 12.5 %, 12.3 %, and 13.3 %. In the first writing-reading cycle for the message "CAMBRIDGE" (Table S5, Supplemental Material), the individual bit-error-rate values for each letter were 12.5 %, 20.0 %, 11.1 %, 10.0 %, 30.0 %, 22.2 %, 33.3 %, 12.5 %, and 10.0 %. The bit error rate can be effectively reduced by aggregating more events during analysis[11].

Chemical-based digital polymers[10] were decoded using MS/MS sequencing. For information stored in short polymer chains, such as an 8-monomer oligo(phosphodiester), full recovery without any error could potentially be achieved.

Redundancy requirements refer to the need to incorporate additional oligos beyond the data payload to ensure reliable data recovery and to provide robustness against decoding errors.

NAPDISS and chemical-based digital polymers[10] involve no non-coding strands, resulting in zero redundancy. For DNA Fountain[6], the reported redundancy is 7%. High-density random-access DNA storage[7] exhibits 15% redundancy for 33 files and 25% redundancy for two files (file number 33 and the metadata). Redundancy-related information is not available for studies on DNA origami cryptography[8] and DNA nanostructure-based hard drives[9].

Information security level is reflected by the encryption scheme, which ensures the confidentiality, integrity, and availability of the target information.

NAPDISS and chemical-based digital polymers[10] utilize photoisomeric DNA and light-cleavable oligo(phosphodiester) as encryption media, supporting multidimensional information encryption by using illumination parameters (e.g., wavelength, intensity, and exposure time) as secret keys. No information encryption scheme was reported in the studies on DNA Fountain or high-density random-access storage. DNA origami cryptography[8] employs a three-layer encryption scheme, comprising dot-pattern encryption as the outer layer, a steganographic intermediate layer, and DNA origami encryption (DOE) as the innermost layer. DNA nanostructure-based hard drives[9] use a two-layer encryption scheme involving biotin-labeled complementary oligos for nanostrucure pattern encryption and streptavidin as a steganographic layer. Streptavidin-biotin binding

enables solid-state nanopore detection of nanostructure-based binary data bits, defined as “0” (without streptavidin) and “1” (with streptavidin) on the DNA hard drive.

References

1. Cao C, Liao D-F, Yu J *et al.* Construction of an aerolysin nanopore in a lipid bilayer for single-oligonucleotide analysis. *Nat Protoc* 2017; **12**: 1901–11.

2. Wu X-Y, Wang M-B, Wang Y-Q *et al.* Precise construction and tuning of an aerolysin single-biomolecule interface for single-molecule sensing. *CCS Chem* 2019; **1**: 304–12.

3. Ying Y-L, Li Z-Y, Hu Z-L *et al.* A time-resolved single-molecular train based on aerolysin nanopore. *Chem.* 2018; **4**: 1893–901.

4. Hu Z-L, Li Z-Y, Ying Y-L *et al.* Real-time and accurate identification of single oligonucleotide photoisomers via an aerolysin nanopore. *Anal Chem* 2018; **90**: 4268–72.

5. Jiang J, Li M-Y, Wu X-Y *et al.* Protein nanopore reveals the renin–angiotensin system crosstalk with single-amino-acid resolution. *Nat Chem* 2023; **15**: 578–86.

6. Erlich Y and Zielinski D. DNA Fountain enables a robust and efficient storage architecture. *Science* 2017; **355**: 950–4.

7. Organick L, Ang SD, Chen Y-J *et al.* Random access in large-scale DNA data storage. *Nat Biotechnol* 2018; **36**: 242–8.

8. Zhang Y, Wang F, Chao J *et al.* DNA origami cryptography for secure communication. *Nat Commun* 2019; **10**: 5469.

9. Chen K, Zhu J, Bošković F *et al.* Nanopore-based DNA hard drives for rewritable and secure data storage. *Nano Lett* 2020; **20**: 3754–60.

10. König NF, Al Ouahabi A, Oswald L *et al.* Photo-editable macromolecular information. *Nat Commun* 2019; **10**: 3774.

11. Chen K, Kong J, Zhu J *et al.* Digital data storage using DNA nanostructures and solid-state nanopores. *Nano Lett* 2019; **19**: 1210–5.
